# Fuzzy Logic, Artificial Neural Network, and Adaptive Neuro-Fuzzy Inference Methodology for Soft Computation and Modeling of Ion Sensing Data of a Terpyridyl-Imidazole Based Bifunctional Receptor

**DOI:** 10.3389/fchem.2022.864363

**Published:** 2022-03-23

**Authors:** Anik Sahoo, Sujoy Baitalik

**Affiliations:** Inorganic Chemistry Section, Department of Chemistry, Jadavpur University, Kolkata, India

**Keywords:** terpyridine, combinatorial logic, keypad lock, fuzzy logic, ANN, ANFIS

## Abstract

Anion and cation sensing aspects of a terpyridyl-imidazole based receptor have been utilized in this work for the fabrication of multiply configurable Boolean and fuzzy logic systems. The terpyridine moiety of the receptor is used for cation sensing through coordination, whereas the imidazole motif is utilized for anion sensing *via* hydrogen bonding interaction and/or anion-induced deprotonation, and the recognition event was monitored through absorption and emission spectroscopy. The receptor functions as a selective sensor for F^−^ and Fe^2+^ among the studied anions and cations, respectively. Interestingly, the complexation of the receptor by Fe^2+^ and its decomplexation by F^−^ and deprotonation of the receptor by F^−^ and restoration to its initial form by acid are reversible and can be recycled. The receptor can mimic various logic operations such as combinatorial logic gate and keypad lock using its spectral responses through the sequential use of ionic inputs. Conducting very detailed sensing studies by varying the concentration of the analytes within a wide domain is often very time-consuming, laborious, and expensive. To decrease the time and expenses of the investigations, soft computing approaches such as artificial neural networks (ANNs), fuzzy logic, or adaptive neuro-fuzzy inference system (ANFIS) can be recommended to predict the experimental spectral data. Soft computing approaches to artificial intelligence (AI) include neural networks, fuzzy systems, evolutionary computation, and other tools based on statistical and mathematical optimizations. This study compares fuzzy, ANN, and ANFIS outputs to model the protonation-deprotonation and complexation-decomplexation behaviors of the receptor. Triangular membership functions (*trimf*) are used to model the ANFIS methodology. A good correlation is observed between experimental and model output data. The testing root mean square error (RMSE) for the ANFIS model is 0.0023 for protonation-deprotonation and 0.0036 for complexation-decomplexation data.

## Graphical Abstract

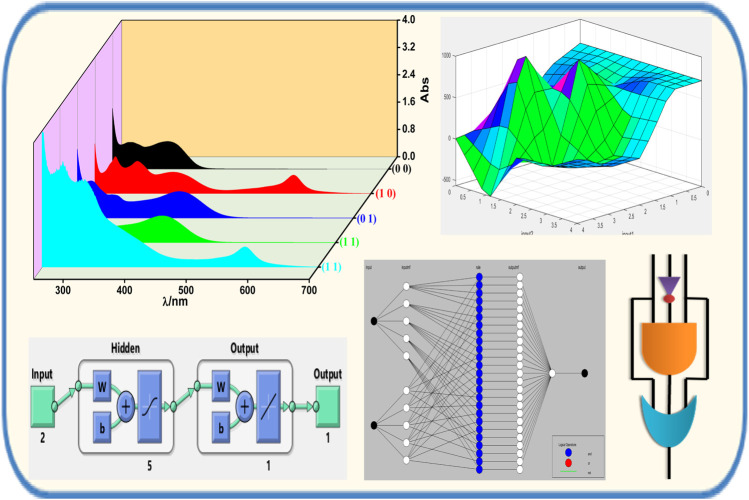

## Introduction

The usage of machine learning (ML) and diverse artificial intelligence (AI) tools (Zadeh, 1973; Szaciłowski, 2008; Zadeh, 2008; Gentili, 2017a; Mater and Coote, 2019; Pflüger and Glorius, 2020; Artrith et al., 2021; He et al., 2021) has been growing enormously in chemistry, biology, and materials sciences. Current research interest is focused mainly on the design of smart materials and the analysis of their physicochemical data (such as sensing, bio-sensing, and imaging) for diagnostic purposes. Little progress has been made in other AI sub-areas, such as fuzzy (Zadeh, 1996; Gentili, 2007; Gentili, 2008; Gentili, 2011; Gentili, 2011; Gentili et al., 2017b, Gentili et al., 2017c; Gentili, 2014; Schumann and Adamatzky, 2015; Gentili et al., 2016; Gentili, 2018), ANNs, ANFIS, robotics, evolutionary computation, and natural language processing and planning (Giri Nandagopal and Selvaraju, 2016; Huang et al., 2012; Razzak et al., 2012; Bingöl et al., 2013; İnal, 2014; Babanezhad et al., 2020a; Babanezhad et al., 2020b; Babanezhad et al., 2020c). Creation of dependable and exhaustive database can extend the ML to a wider domain of application. Much effort is now being given to prosper the AI with vague and imprecise inputs. The function in Boolean logic (BL) (de silva et al., 1993; Ling et al., 2015; de Silva and McClenaghan, 2004; de Silva., 2011; de Silva et al., 2000; Szaciłowski, 2004; Szaciłowski et al., 2006; Adamatzky and Costello, 2002; Adamatzky et al., 2020; Gale et al., 2013; Adamatzky et al., 2016) relies on stretching the output signal in between the two extremes of “0” and “1”. However, most real systems are composed of many intermediate states. The fuzzy logic (FL) is believed to be a probable alternative to BL in identifying the intermediate states. The motivation in choosing FLS relies on the motivation that thought and the decision-making process in humans is extremely complicated to be precisely defined and believed to function as an automatic fine-controlling administer for an innumerable number of intervening steps with a varying degree of truths. FLS consists of nonlinear scaling of the input vectors to the scalar outputs. The number of molecular systems implementing the FLS is relatively sparse in the literature.

**CHART 1:**
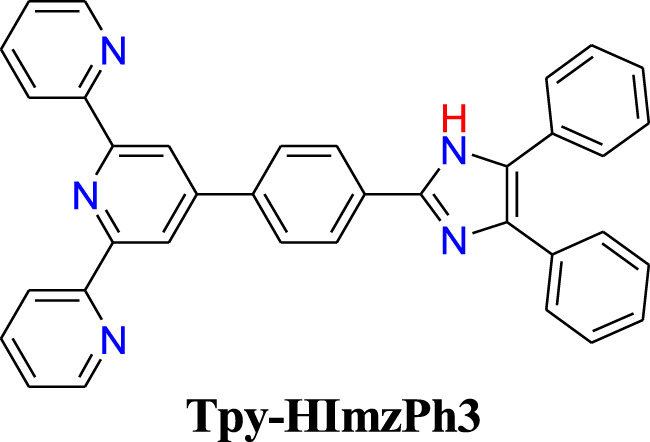
Chemical structure of the terpyridyl-imidazole based receptor.

In this work, we have utilized our previously reported terpyridyl-imidazole system (tpy-HImzPh_3_) (Bhaumik et al., 2011), wherein a terpyridine moiety capable of coordinating with several bivalent 3d metals is covalently coupled with a triphenyl-imidazole motif capable of interacting with selected anions (**Chart 1**) (Bhaumik et al., 2011; Karmakar et al., 2014; Mondal et al., 2017; Karmakar et al., 2015a; Mukherjee et al., 2021). Using its absorption and emission spectral responses as a function of a specific set of cations and anions, multiple Boolean logic (BL) functions such as combinatorial logic of AND, OR, and NOT gates (Omana et al., 2003; Zhang et al., 2015; Magri and Spiteri, 2017; Goldsworthy et al., 2018) and molecular level keypad lock are demonstrated (Margulies et al., 2007; Strack et al., 2008; Andrasson et al., 2009; Kumar et al., 2009; Bhalla and Kumar, 2012; Zou et al., 2012; Jiang and Ng, 2014; Carvalho et al., 2015; Chen et al., 2015; Karmakar et al., 2015b; Mondal et al., 2015). Herein, we also executed fuzzy logic for creating an infinite-valued logic scheme using the emission spectral output upon the action of specific cations (H^+^ and/or Fe^2+^) and anion (F^−^).

ANNs are biologically motivated systems comprised of extensively connected processing elements arranged in layers and bound with weighted interrelations. Usually, ANN is framed by a numerical learning algorithm and could be “trained” to approximate effectively any nonlinear function to a required degree of accuracy. To this end, ANN is believed to be a universal approximator class. We have designed two ANN models based on reversible deprotonation-protonation induced by anions and acid and recomplexation-decomplexation behavior of the receptor in the presence of M^2+^ and F^−^ ions. Due to the lack of learning capability of fuzzy model and paucity of transparency of the ANN model, we also implemented the neuro-fuzzy system, which represents a type of hybrid intelligent system amalgamating the principle features of ANN and fuzzy logic. The objective is to eliminate the difficulty of implementing fuzzy logic through numerical knowledge or, contrarily, implementing ANN *via* linguistic information. Importantly, we also compared the outcomes of the fuzzy, ANN, and ANFIS methods with the experimental outputs to better model the deprotonation-protonation and complexation-decomplexation behavior of the receptor.

## Results and Discussion

### Overview of the Anion and Cation Sensing Behavior of the Receptor

The method of synthesis, thorough characterization, and anion and cation sensing properties of tpy-HImzPh_3_ was previously reported by our group (Bhaumik et al., 2011). A brief overview of the ion sensing behavior of the receptor is summarized for the benefit of the readers. Tpy-HImzPh_3_ shows two intense bands. The lower-energy band at 340 nm is due to imidazole→tpy intra-ligand charge transfer (ILCT) transition, whereas the higher-energy band at 285 nm is due to π–π* transition in DMF-MeCN (1:9, v/v) solution. The receptor also exhibits a strong emission band at 485 nm with a quantum yield (Φ) of 0.095 and a lifetime (τ) of 2.55 ns. The anion and cation sensing behavior of tpy-HImzPh_3_ was studied in DMF-MeCN (1:9, v/v) solution through absorption and emission spectroscopic techniques. The receptor functions as a selective sensor for F^−^ and Fe^2+^ among the studied anions and cations, respectively. The spectral change upon incremental addition of F^−^ and Fe^2+^ is displayed in Supplementary Figure S1. The absorption peak at 341 nm gets diminished systematically, accompanied by an increase in the new band at 420 nm and the evolution of bright yellow color upon gradual addition of F^−^. The bathochromic shift is probably because of F^−^-induced deprotonation of the NH motif, which enhances the electron density at the imidazolate moiety and facilitates the electron transfer process. In contrast, the addition of Fe^2+^ leads to generation and gradual intensification of the peak at 575 nm with the evolution of a violet color, and saturation occurs with 0.5 equiv. Fe^2+^. The violet color is due to Fe(d)→tpy (π^*^) MLCT transition in the resulting [Fe(tpy-HImzPh_3_)_2_]^2+^ complex. Complete quenching of emission of the receptor is observed in the presence of both Fe^2+^ and F^−^ ions. It is to be noted that, upon excitation at 575 nm, the Fe(II) complex does not show any emission band due to the presence of low-lying triplet/quintet metal-centered (^3/5^MC) excited states.

Interestingly, the complexation of tpy-HImzPh_3_ by Fe^2+^ and its decomplexation by F^−^ are reversible and can be repeated many times. Similarly, the deprotonation of the receptor by F^−^ and reverting into its initial protonated form by acid is also reversible and can be recycled many times (Supplementary Figure S1). The reversible deprotonation-protonation and complexation-decomplexation behavior of the receptor has been employed for the construction of different types of logic devices. The next section shows that the receptor can mimic various logic operations using its spectral responses through the sequential use of ionic inputs.

### Combinatorial Logic System

**FIGURE 1:**
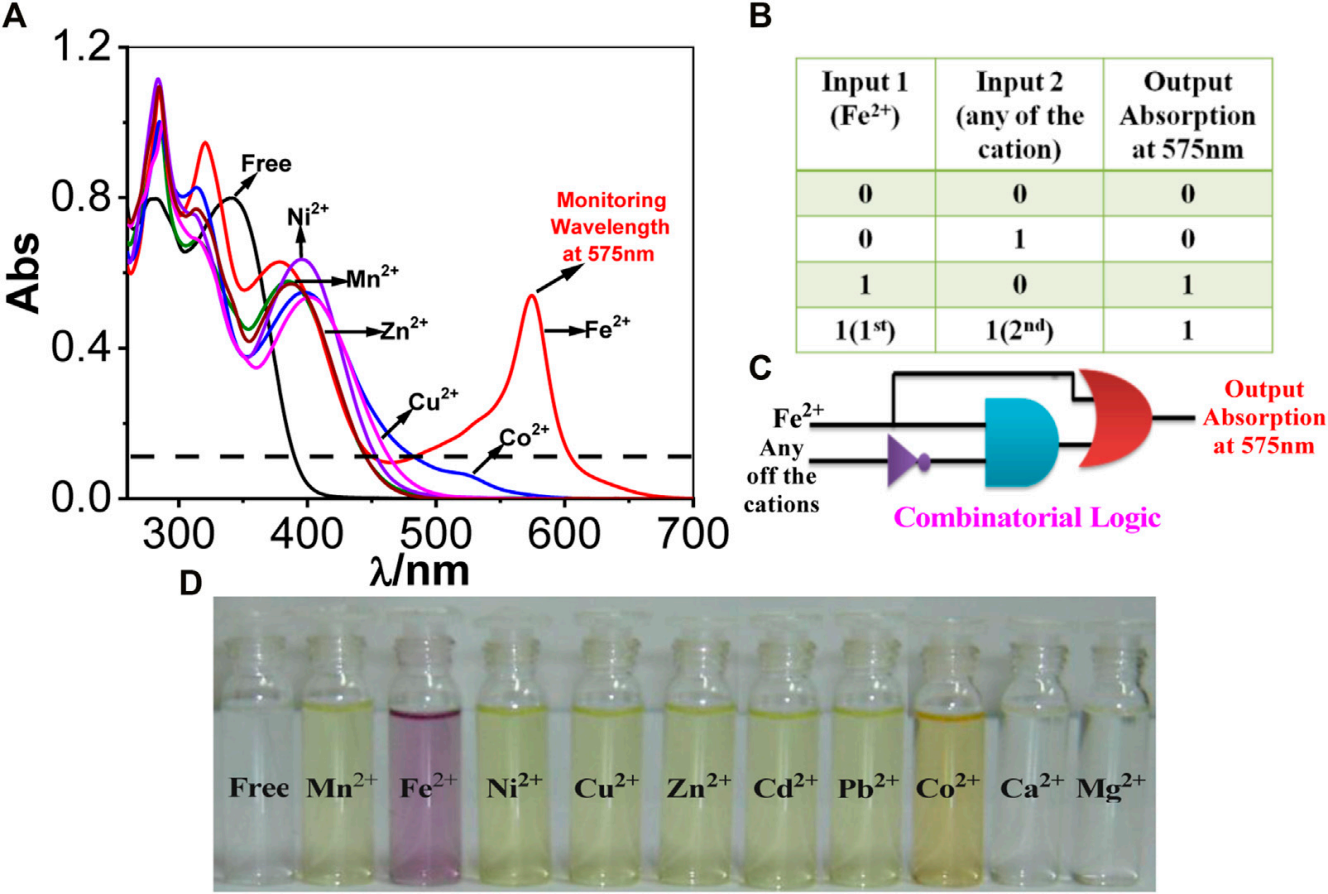
**(A)** UV-Vis absorption spectrum of tpy-HImzPh_3_ in the presence of different cations. **(B)** Truth table of the combinatorial logic system. **(C)** Schematic diagram of the combinatorial logic system. **(D)** Visual color changes in the presence of various cations.

It is a type of digital logic that is implemented by Boolean circuits. In this section, we utilize the spectral response of the tpy-HImzPh_3_ upon the action of Fe^2+^ as input 1, and the rest of the studied bivalent cations can be treated as input 2. Among the studied cations, only Fe^2+^ can induce a strong absorption band at 575 nm which is well above the threshold energy level and gives rise to the “ON” state 1 (Figure 1). Based on the absorption spectral behavior of tpy-HImzPh_3_ upon the influence of different cations and monitoring the signal at 575 nm, the function of a combinatorial logic system can be mimicked.

**FIGURE 2:**
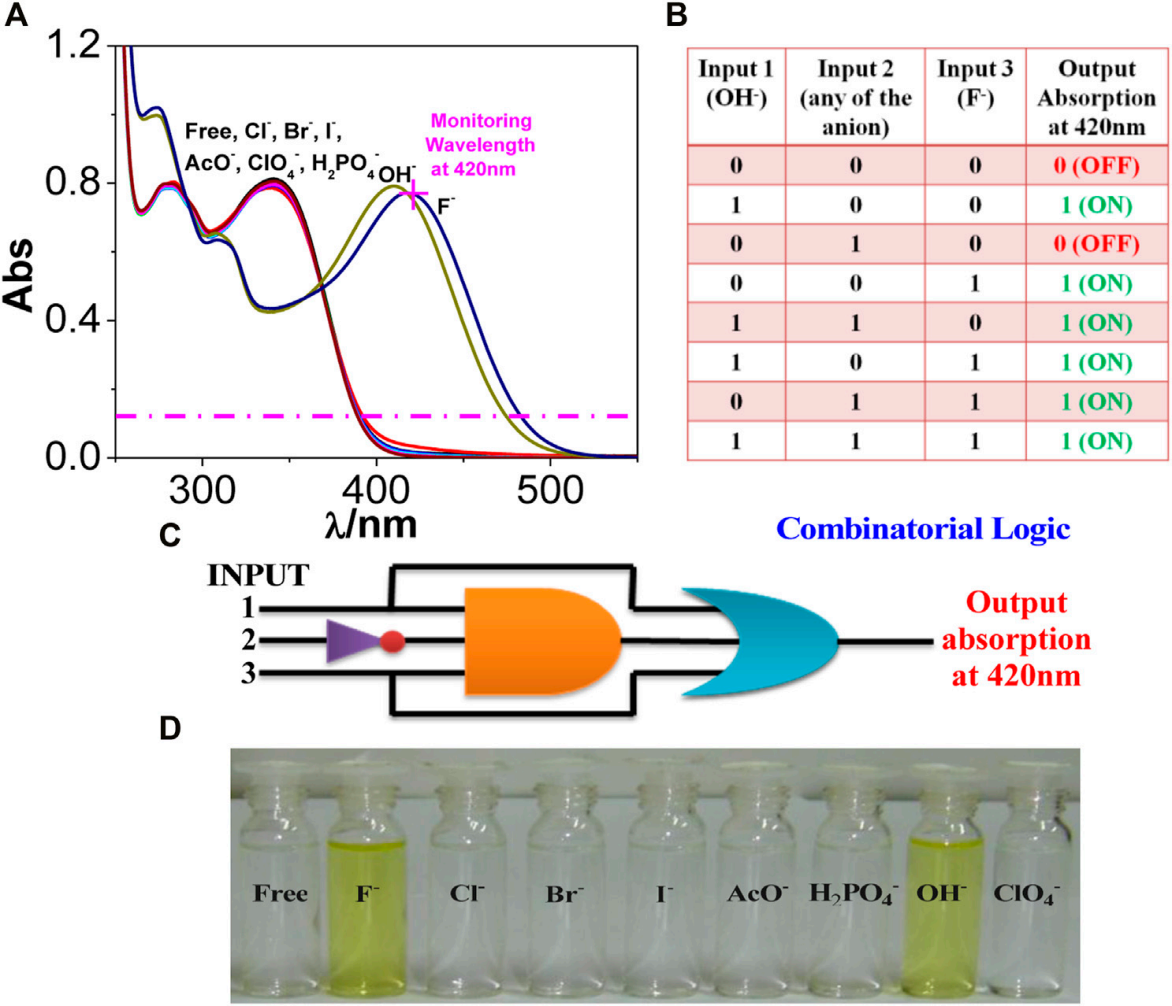
**(A)** UV-Vis absorption spectrum of tpy-HImzPh_3_ in the presence of different anions. **(B)** Truth table of the combinatorial logic system. **(C)** Schematic diagram of the combinatorial logic system. **(D)** Visual color changes in the presence of different anions.

**FIGURE 3:**
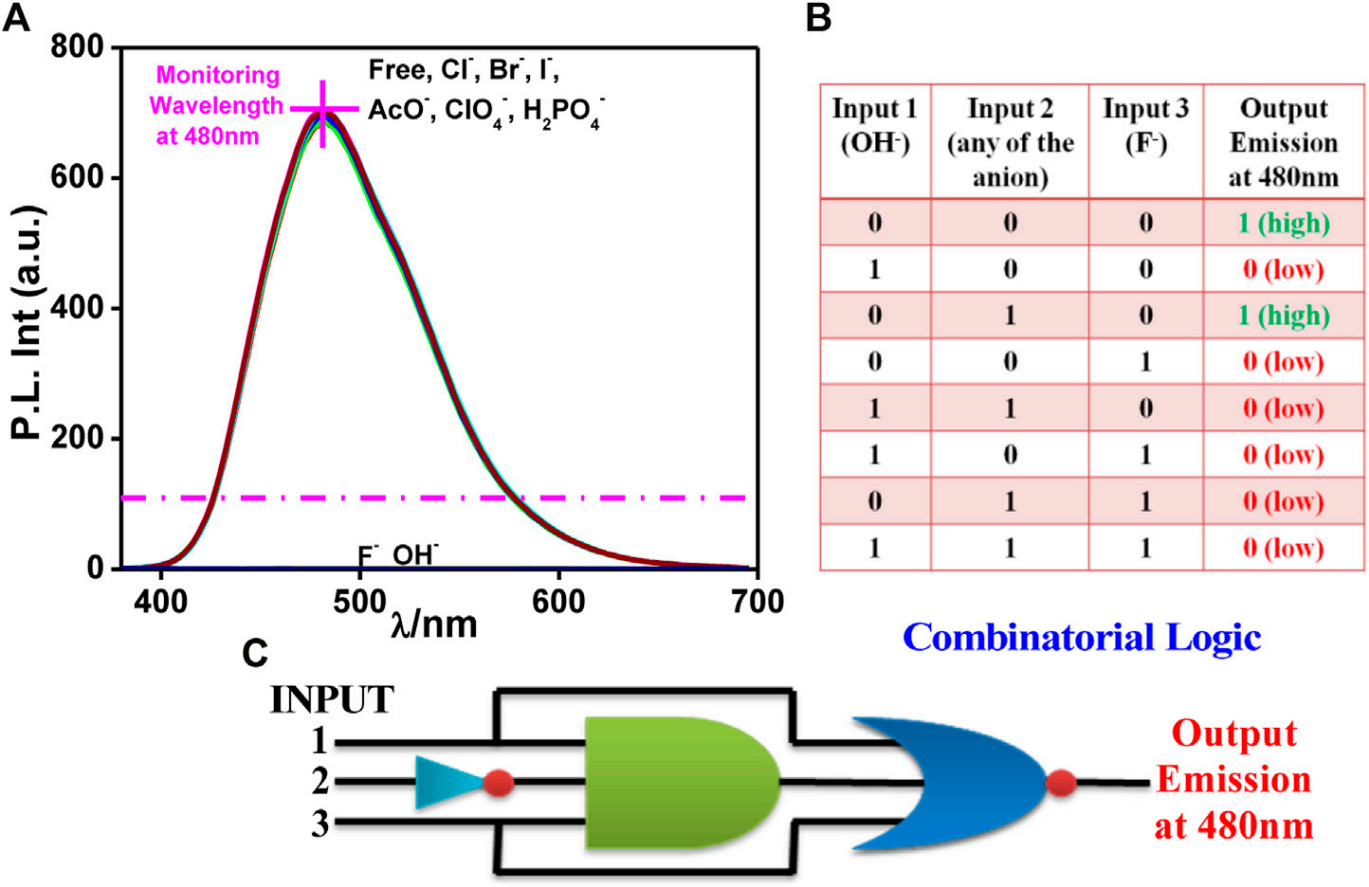
**(A)** Photoluminescence spectrum of tpy-HImzPh_3_ in the presence of different anions. **(B)** Truth table of the combinatorial logic system. **(C)** Schematic diagram of the combinatorial logic system.

Two other combinatorial logic functions can also be mimicked by utilizing the spectral outputs in the presence of different anions. Among the studied anions, only OH^−^ (input 1) or F^−^ (input 3) leads to the evolution of the absorption maximum at 420 nm above the threshold level and thus corresponds to the ON state (Figure 2A). In contrast, the remaining anions (input 2) correspond to the OFF state. By contrast, OH^−^ (input 1) or F^−^ (input 3) induces complete quenching of emission displaying the OFF state, whereas the remaining anions, which are unable to quench the emission intensity, correspond to the ON state (Figure 3A). The output arising from the different possible combinations of inputs are provided in the truth table of Figures 2B, 3B To better understand the functions of anions in UV and photoluminescence property of tpy-HImzPh, a schematic diagram of combinational logic circuits is presented Figure 2C and Figure 3C. The visual colour change in presence of different anions is also displayed in Figure 2D.

### Keypad Lock

**FIGURE 4:**
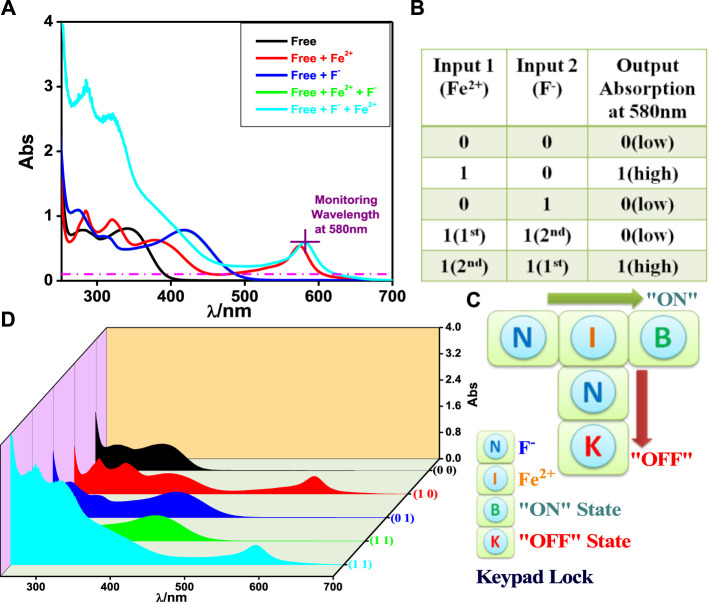
**(A)** UV-Vis absorption spectrum of tpy-HImzPh_3_ upon interaction with F^−^ and Fe^2+^. Truth table and schematic display of the security keypad lock **(B,C)**. **(D)** 3D display of the variation of absorbance in the presence of the inputs.

The absorbance at 580 nm is used as the output signal upon the influence of Fe^2+^ (input 1) and F^−^ (input 2) for this purpose. In Figure 4A, the input Fe^2+^ is earmarked as “**I**,” whereas F^−^ is allocated as “**N**.” “**B**” and “**K**” correspond to the “ON state” and “OFF state,” respectively. There is no absorption above the threshold energy level at 580 nm in the absence of both inputs implying the “OFF state.” The addition of “**N**” followed by “**I**” induces enhancement of absorption above the threshold level, leading to the “ON state” and creating a secret password “**NIB**.” Reversing the sequence of addition (“**I**” followed by “**N**”) induces a remarkable decrease in emission below the threshold indicating the “OFF state” and leads to the creation of the password “**INK**,” which cannot unlock the keypad lock. Thus, only the authorized person can unlock it. It is a novel approach to protecting information at the molecular level and much better than the common number-based PIN (Figure 4).

### Fuzzy Logic Operations

In Boolean systems, we use crisp values that define a strict boundary, either true (1) or false (0). They are unable to define any intermediate values. In the real world, we often encounter a situation where we cannot confidently determine whether the state is true (1) or false (0). The fuzzy logic, first proposed by Lotfi Zadeh in 1965 (Zadeh, 1996), provides an easy alternative to this end and some flexibility in reasoning. Due to the unclarity and vagueness of most chemical reactions, the computation based on fuzzy logic is assumed to be a probable substitute to tackle the indecisive information in the domain of the binary logic scheme.

**FIGURE 5:**
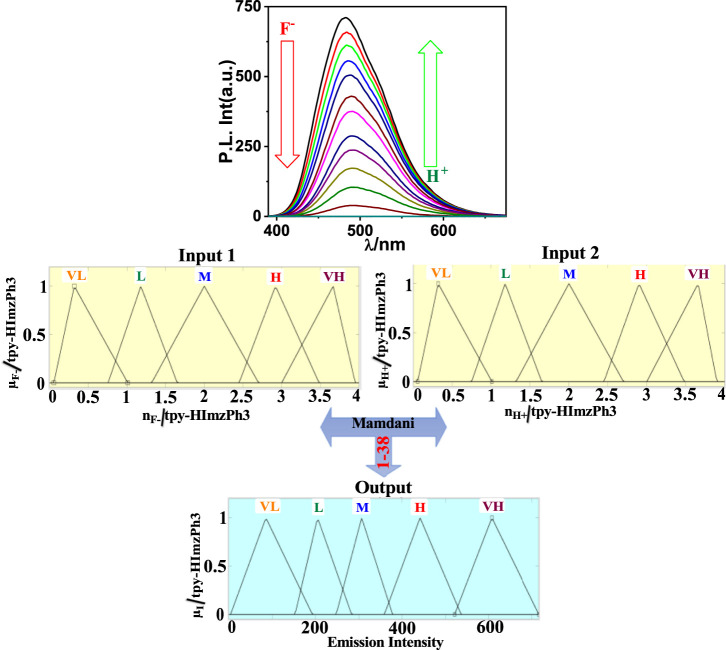
Schematic display of fuzzy logic scheme based on fuzzy inference rules upon monitoring the emission intensity as a function of F^−^ and H^+^. Fuzzy variables are decomposed in five fuzzy sets. F^−^: (1) very low [trimf μ_verylow_, (0.056 0.32 1.01)]; (2) low [trimf μ_low_, (0.757 1.18 1.65)]; (3) medium [trimf μ_medium_, (1.32 2 2.72)]; (4) high [trimf μ_high_, (2.45 2.92 3.482)]; and (5) very high [trimf μ_veryhigh_, (3 3.665 3.95). H^+^: (1) (1) very low (trimf μ_verylow_, (0.056 0.32 1.01)]; (2) low [trimf μ_low_, (0.757 1.18 1.65)]; (3) medium [trimf μ_medium_, (1.32 2 2.72)]; (4) high [trimf μ_high_, (2.45 2.92 3.482)]; and (5) very high [trimf μ_veryhigh_, (3 3.665 3.95). Emission intensity (output): (1) very low [trimf μ_verylow_, (4.28 85.8 193)]; (2) low [trimf μ_low_, (153 205 283.5)]; (3) medium [trimf μ_medium_, (248 306.7 378)]; (4) high [trimf μ_high_, (359 441.4 536)]; and (5) very high [trimf μ_veryhigh_, (521 607 716.3)].

**FIGURE 6:**
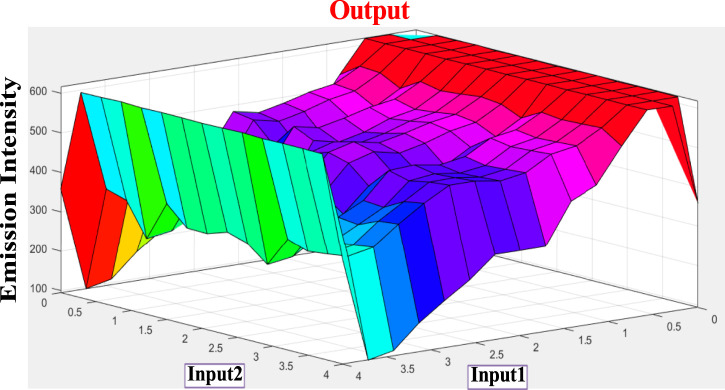
3D display of the dependence of emission intensity of tpy-HImzPh_3_ at 485 nm upon the action of F^−^ and H+.

As shown in Figure 5, the spectral change of tpy-HImzPh_3_ greatly varies upon the action of F^−^ (input 1) and H^+^ (input 2). Instead of its indefinite character and large degree of change, the present system’s variables can be disclosed in terms of five lingual parameters of the triangular molecular functions (*trimf*): very low, low, medium, high, and very high (Mamdani, 1977). The influence of the varying amount of H^+^ and F^−^ on the emission intensity of tpy-HImzPh_3_ could be presented in the form of fuzzy sets (Figure 5). An assortment of divergent IF-THEN statements involving the inference rules is provided in Supplementary Table S1. The IF-portion conforms to the antecedent, whereas the THEN-portion correlates to the consequence. The quenching of emission occurs in the presence of F^−^, whereas the regeneration of emission takes place upon the action H^+^. To this end, fuzzy logic is applied to tpy-HImzPh_3_ upon monitoring the emission intensity with changing concentrations of H^+^ and F^−^ inputs (Supplementary Table S2). The feasible consolidation of F^−^ and H^+^ generates 38 rules (Supplementary Table S1 and Supplementary Figure S2). Furthermore, the variation of emission intensity upon combined actions of H^+^ and F^−^ is portrayed in a 3D plot (Figure 6).

### Artificial Neural Network

**FIGURE 7:**
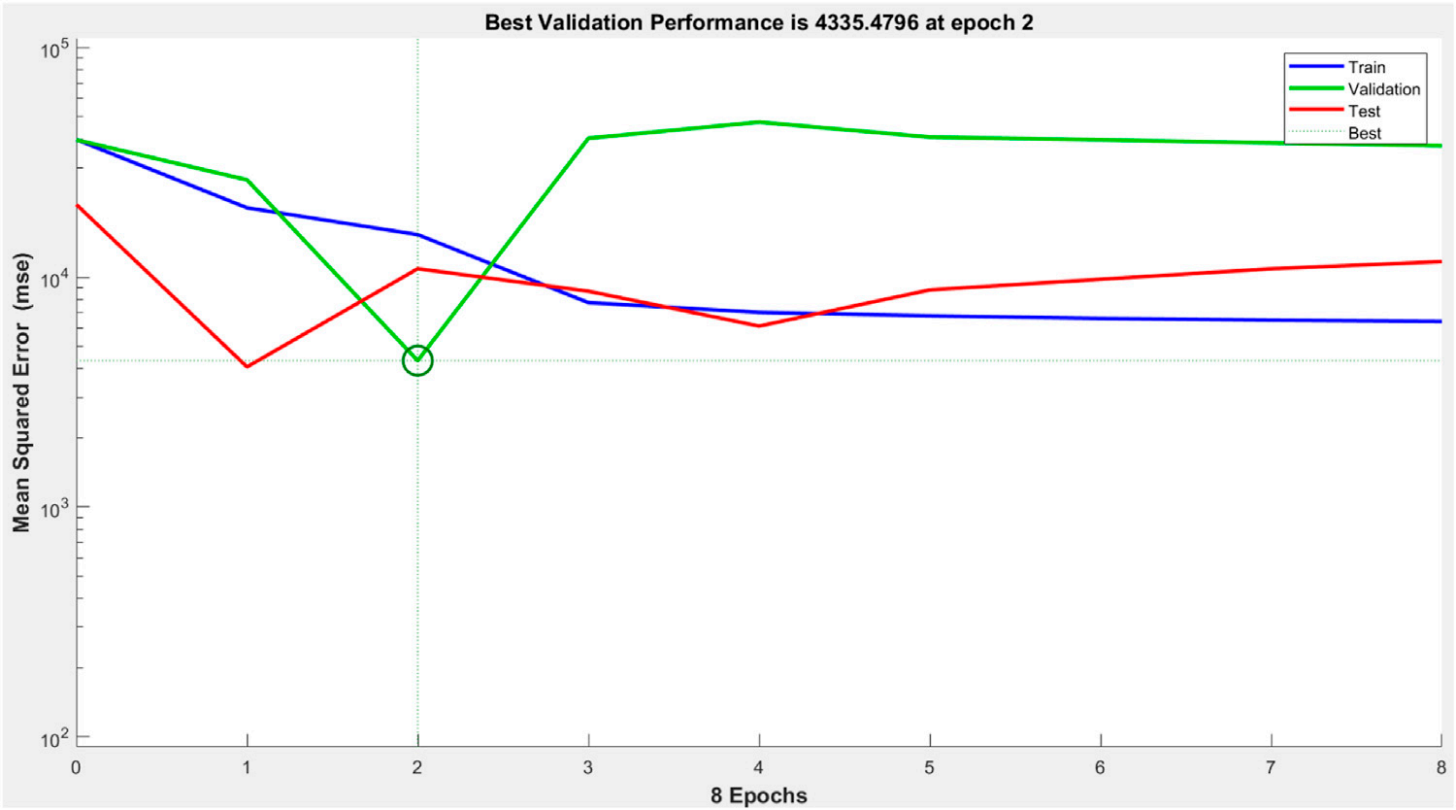
The performance of the designed ANN model.

Fuzzy logic has good knowledge representation ability but weak learning capability. To this end, we tried to formulate the ANN mathematical algorithm and modeling method that correlates the input and output dependence of the receptor. ANN is a powerful aid for modeling the nonlinear functions, which represent real-world systems. ANN is constructed *via* the compilation of artificial neurons, which mirror the connectedness of neurons in the human brain to carry out a task with enhanced performance *via* learning, training, and continuous improvement. We used the Levenberg–Marquardt algorithm for training purposes. Input data present the network, and target data define the desired network output. Supplementary Table S2 represents the emission outputs upon the action of 25 different combinations of two inputs (input 1 = F^−^ and input 2 = H^+^). Thus, the 25 × 2 matrix represents the static input data of 25 samples involving two inputs, whereas the 25 × 1 matrix represents the static output data of one element. Now, the 25 samples are divided into three data sets. 70% of data are conferred for the training, and the network is corrected according to its error. 15% of data are employed to compute the network generalization and halt training. When generalization stops improving, data validation takes place. The remaining 15% of data provide an independent measure of the network performance during and after the training, called testing data (Figure 7).

**FIGURE 8:**
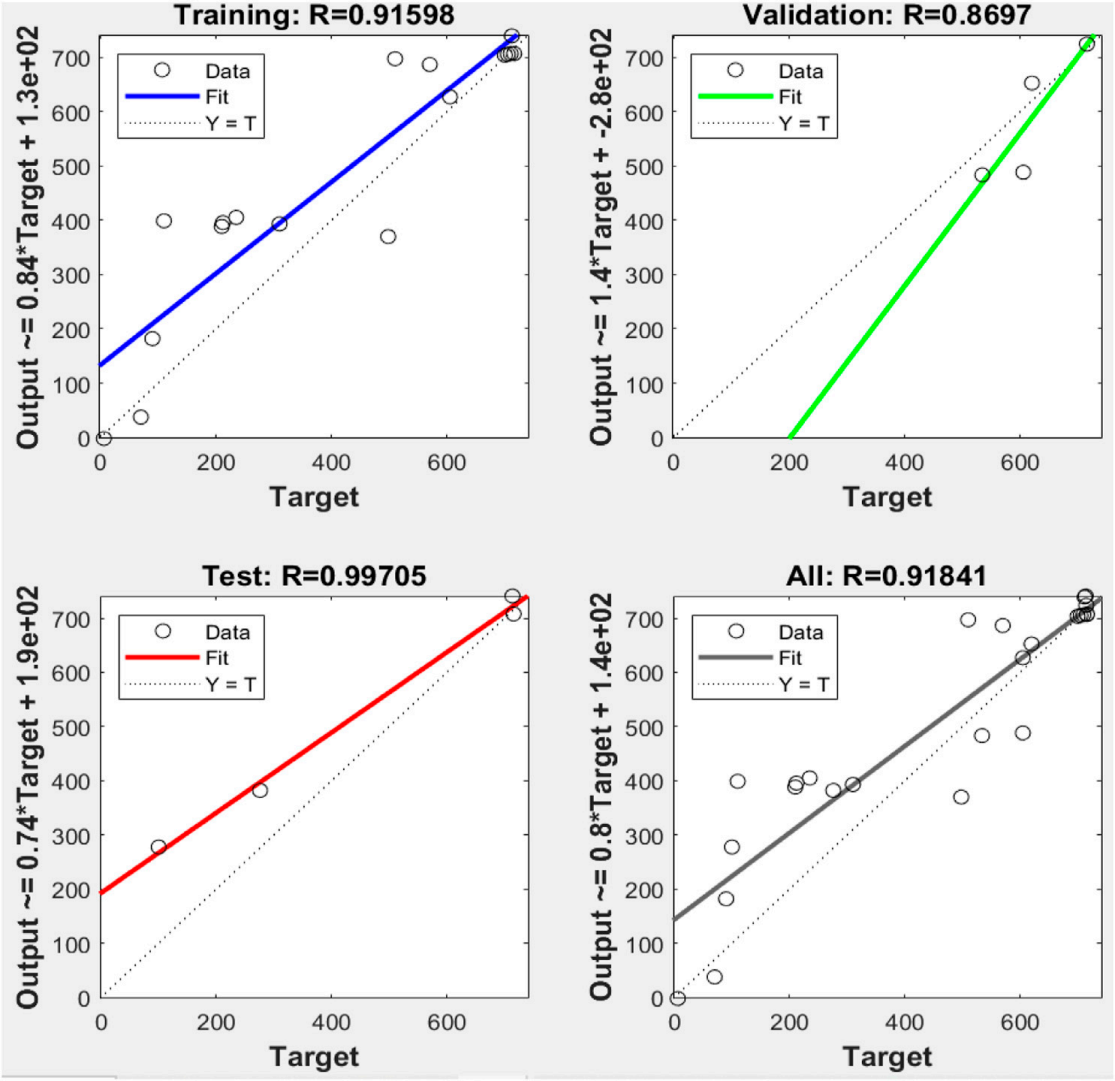
Comparison between linear regression and ANN model results plotted and the observed values for training, validation, and testing.

It clearly shows that the model’s best validation performance is 4335.47 at epoch 2. The enhancement of the green-colored line after epoch 2 suggests that the increment of the mean square error (MSE) and training is halted. The regression values (R) measure the correlation between the outputs and targets. The R values close to 1 imply a close relationship between output and targets and very good performance of the model (Figure 8). The training state of the ANN model up to epoch 8 is given in Supplementary Figure S3.

**FIGURE 9:**
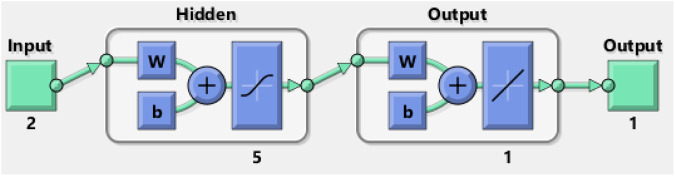
Artificial neural network model consisting of two inputs, five hidden layers, and one output.

The bins are the number of vertical bars on the graph (Supplementary Figure S4). The *y*-axis designates the number of samples in the database, which exit in a particular bin, for example, at the middle of the plot, the bin corresponding to the error of −7.425 to 13.42. The height of that bin for the training data set lies below but close to 2, and that for the validation data set varies between 2 and 3. In the present case, the zero error point is situated under the bin with the center at −7.425. The total error from neural network ranges from −278.5 (leftmost bin) to 117.7 (rightmost bin). The error histogram represents the histogram of the errors between target values and predicted values after training a feed-forward neural network. As the error values suggest how predicted values deviate from the target values, this could be negative. This error range is spitted up into 20 smaller bins, so each bin has a width of [117.7-(−278.5)]/20 = 19.81 (Supplementary Figure S4). There are three layers: input, hidden, and output. Each hidden layer performs a nonlinear transformation of the inputs entered into the network. Inputs are loaded into the input layer, and each node gives rise to an output value through an activation function. The outputs of the input layer again act as the inputs to the next hidden layer (Figure 9).

On putting the different input values in the rule viewer of fuzzy logic and the command section of ANN model in MATLAB R2018a, we obtain the output values presented in Table 1, which indicate that the difference between experimental and fuzzy logic output is greater than the difference between experimental and ANN model output because of the neural network’s inability to explain the decision (lack of transparency) and fuzzy logic’s weakness of learning.

**TABLE 1:** Experimental, fuzzy, and ANN model data in the presence of different input combinations.

Input 1 (F)	Input 1 (H*)	Experimental output data	Data output based on fuzzy logic	Data output based on ANN model
1	1	620	580	652
4	4	212	400	395
4	0	6	35	1
1	3	700	680	703
1	2	510	390	697

### Adaptive Neuro-Fuzzy Inference System

**FIGURE 10:**
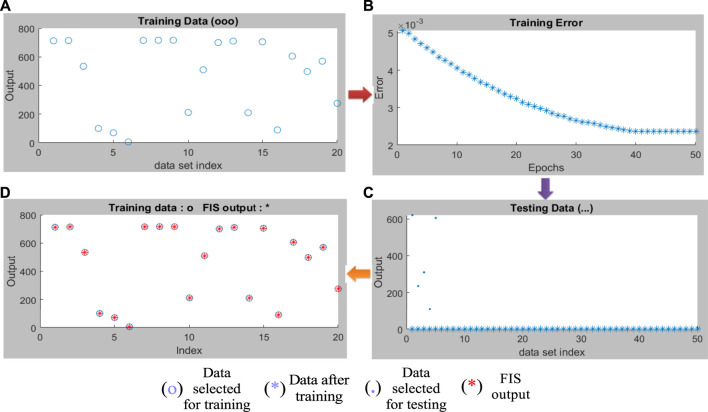
**(A)** Selected training data to design the ANFIS model. **(B)** Training error minimization up to 50 epochs. **(C)** Combination of training and testing data. **(D)** Compilation of testing data and FIS output.

**FIGURE 11:**
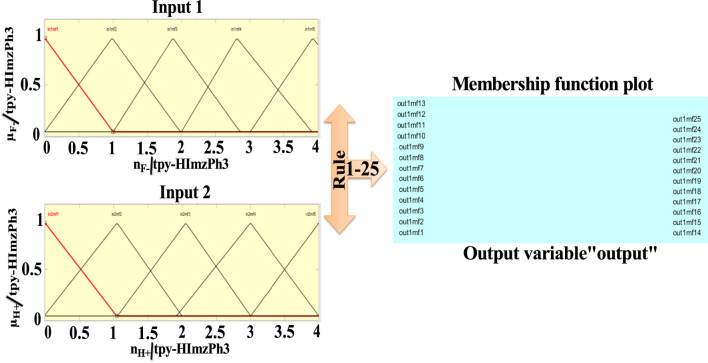
Schematic presentation of ANFIS based on Sugeno’s method maintaining 25 rules.

**FIGURE 12:**
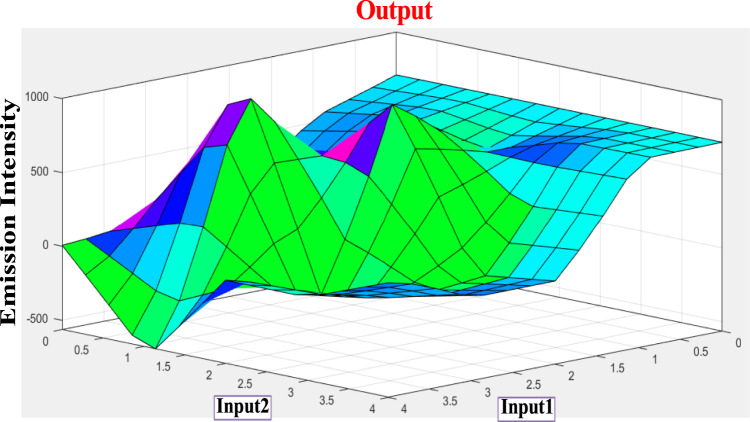
Three-dimensional representation (based on Sugeno’s method) of the dependence of emission intensity of tpy-HImzPh_3_ at 485 upon simultaneous action of two inputs (F^−^ and H^+^).

Combining the fuzzy and neural network overcomes the drawback of individual ones (Sugeno and Yasukhiro, 1993; Jang and Sun, 1995). Robustness, solidity, and high generalization capability of the ANFIS model provide room for applications that involve crisp inputs and outputs. To develop the system, we have used 70% of the data for training purposes and the remaining 30% for testing (Figure 10A,C,D). Figure 10B shows that the training error is reduced every time up to 50 epochs, indicating that the system is learning in every single step. Due to the presence of two inputs and five membership functions each, the system will generate 5^2^ = 25 rules (Supplementary Table S3 and Supplementary Figure S5). The feasible consolidation of F^−^ and H^+^ generates 25 rules on the basis of Sugeno’s method (Figure 11). On running the generated ANFIS on MATLAB-R2018a and upon commanding the system with different input values, the obtained outputs are summarized in Table 2. Furthermore, the variation of emission intensity upon combined operation of F^−^ and H^+^ is portrayed in a 3D plot (Figure 12).

**TABLE 2:** Experimental and ANFIS generated outputs.

Input 1 (H^+^)	Input 1 (F^-^)	Experimental output data	Data output based on ANFIS logic
1	1	620	618
4	4	212	211
4	0	6	8
1	3	700	700
1	2	510	530

**FIGURE 13:**
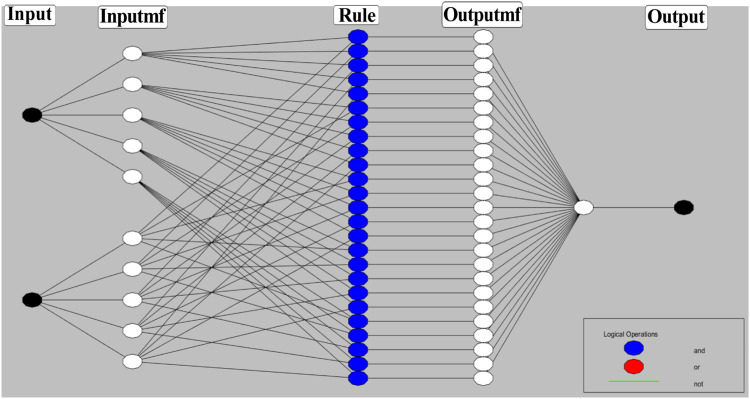
Generated ANFIS structure based on 25 rules.

**FIGURE 14:**
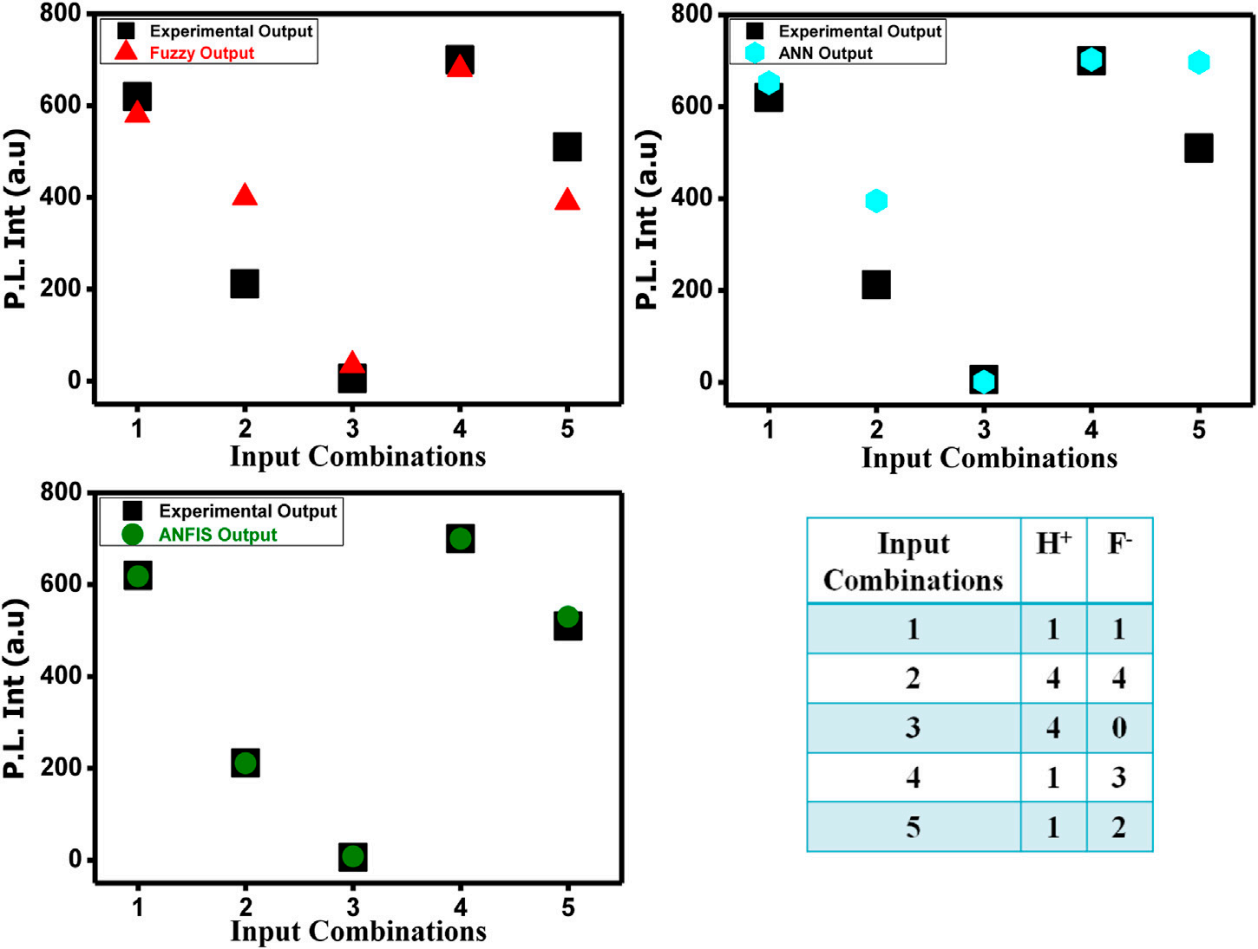
Comparison between experimental emission output data and fuzzy, ANN, and ANFIS output data.

The performance of the ANFIS models in the present study is statically measured by root mean square error (RMSE). The testing RMSE value for this model is 0.0023, suggesting that the model is working properly. We can see that the ANFIS generated output values are closer to the experimental outputs. Therefore, it is a more accurate optimization system than the fuzzy and neural network system. On the basis of 25 rules, we have constructed the ANFIS structure (Figure 13). The comparison and the deviation of the experimental data to those of fuzzy, ANN, and ANFIS outputs are presented in Figure 14.

**FIGURE 15:**
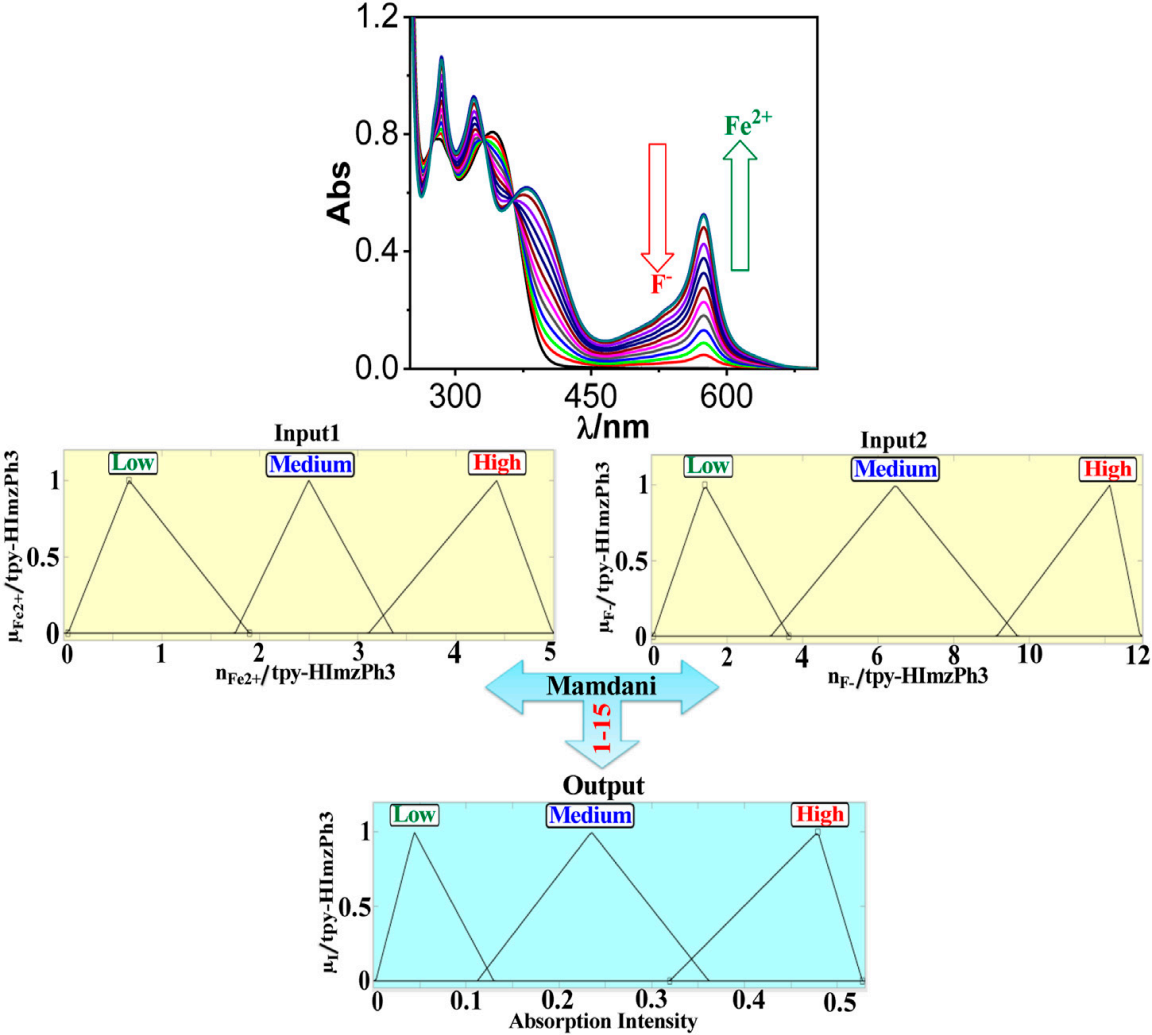
Schematic display of fuzzy logic based on fuzzy inference rules by monitoring absorbance at 575 nm upon the action Fe^2+^ and F^−^ as inputs. Fuzzy variables are decomposed into three fuzzy sets. Fe^2+^: (1) low [trimf μ_low_, (0.042 0.665 1.89)]; (2) medium [trimf μ_medium_, (1.747 2.51 3.36)]; and (3) high [trimf μ_high_, (3.109 4.419 4.989)]. F^−^: (1) low [trimf μ_low_, (0.0426 1.41 3.633)]; (2) medium [trimf μ_medium_, (3.16 6.46 9.69)]; and (3) high [trimf μ_high_, (9.146 12.14 13.01)]. Absorption intensity (output): (1) low [trimf μ_low_, (0.00253 0.0439 0.1297)]; (2) medium [trimf μ_medium_, (0.112 0.235 0.361)]; and (3) high [trimf μ_high_, (0.319 0.479 0.527)].

Fe^2+^ addition causes absorbance enhancement at 575 nm of tpy-HImzPh_3_ (due to complexation), whereas F^−^ causes absorbance depletion (because of decomplexation). We implement fuzzy logic to the receptor upon changing the concentrations of Fe^2+^ and F^−^ ions and by monitoring the absorption spectral response (Supplementary Table S4). We have taken three triangular membership functions (*trimf*) for each input and output. The feasible consolidation of Fe^2+^ and F^−^ generates 15 rules (Figure 15, Supplementary Figure S6, and Supplementary Table S5). Furthermore, the variation of absorption intensity upon the combined operation of Fe^2+^ and F^−^ is portrayed in a 3D plot (Supplementary Figure S7).

### Artificial Neural Network

We also used here the Levenberg–Marquardt algorithm for training purpose. The input data present the network, and the target data define the desired network output. Input 16 × 2 matrix represents static data of 16 samples of 2 inputs and output 16 × 1 matrix represents static data of 16 samples of 1 element. Sixteen samples are divided into three data sets. 70% of data (12 samples) are fed to the network during training, and the network is optimized according to its error. 15% (two samples) of data are used to measure network generalization and halt training, whereas the remaining 15% (two samples) of data do not affect training. However, they give an independent measure of network performance during and after training (Supplementary Figure S8).

Supplementary Figure S8 clearly shows that the model’s best validation performance is 0.0005813 at epoch 14. The enhancement of green-colored spectra after epoch 14 suggests the increment of mean square error (MSE) and training is halted. Regression (R) values measured the correlation between outputs and targets. The R values close to 1 imply a close relationship between output and targets and very good performance of the model (Supplementary Figure S9). The training state of the ANN model up to epoch 20 is given (Supplementary Figure S10). The *y*-axis designates the number of samples from the database, which lies in a particular bin, for example, at the middle of the plot, the bin corresponding to the error of −0.00186 to 0.00214. The height of that bin for the training data set lies below but close to 5, and that of the validation data set varies between 5 and 6. In the present case, the zero error point is situated under the bin with the center at −0.00186 (Supplementary Figure S11). The total error from the neural network ranges from −0.03387 (leftmost bin) to 0.04215 (rightmost bin). This error range is divided into 20 smaller bins, so each bin has a width of [0.04215-(−0.03387)]/20 = 0.0038 (Supplementary Figure S11). As discussed earlier, the three layers are presented in Supplementary Figure S12. On putting the different input values in the rule viewer of fuzzy logic and the command section of the ANN model in MATLAB R2018a, we got the following output values (Table 3).

**TABLE 3:** Experimental, fuzzy, and ANN model data in the presence of different combinations of inputs.

Input 1 (Fe^2+^)	Input 1 (F^-^)	Experimental output data	Data output based on fuzzy logic	Data output based on ANN model
2	6	0.22	0.153	0.207
1	5	0.24	0.059	0.196
5	9	0.15	0.064	0.186
5	0	0.53	0.265	0.496
3	7	0.19	0.161	0.196

### Adaptive Neuro-Fuzzy Inference System

To develop the system, we have used 70% of the data for training purposes and the remaining 30% for testing. Supplementary Figure S14 shows that the training error is reduced every time to 50 epochs, indicating that the system is learning in every single step. Due to the presence of two inputs and three membership functions each, the system will generate 3^2^ = 9 rules (Supplementary Table S6 and Supplementary Figure S13). The plausible compilation of Fe^2+^ and F^−^ generates nine rules on the basis of Sugeno’s method (Supplementary Figure S15). On running the generated ANFIS on MATLAB-R2018a and commanding the system with different input values, we got the following outputs (Table 4). The variation of absorption intensity upon combined operation of Fe^2+^ and F^−^ is shown in a 3D plot (Supplementary Figure S16).

**TABLE 4:** Experimental and ANFIS generated outputs.

Input 1 (H^+^)	Input 1 (F^-^)	Experimental output data	Data output based on ANFIS logic
2	6	0.22	0.211
1	5	0.24	0.241
5	9	0.15	0.155
5	0	0.53	0.531
3	7	0.19	0.192

**FIGURE 16:**
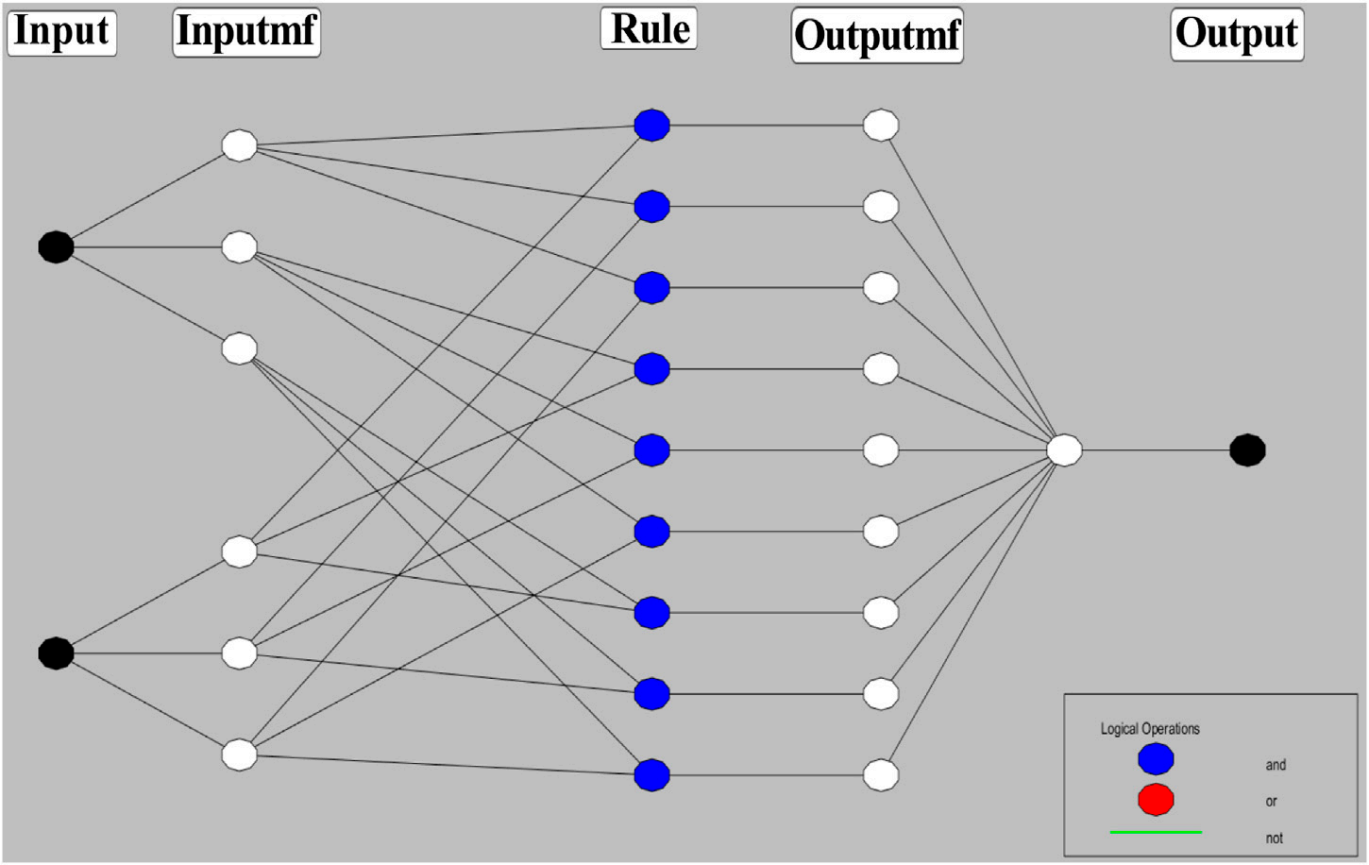
Generated ANFIS structure based on nine rules.

The testing root mean square error (RMSE) for this model is 0.0036, suggesting that the model is working properly. We can see that the ANFIS generated output values are closer to the experimental outputs. Therefore, it is a more accurate system than fuzzy and neural network system. We have constructed the ANFIS structure on the basis of the nine rules (Figure 16). Supplementary Figure S17 shows the deviation between the experimental and fuzzy, ANN, and ANFIS outputs.

## Conclusion

Concerning our recent interest in process information at the molecular level, we a terpyridyl-imidazole based receptor (tpy-HImzPh_3_), which, upon interaction with specific cations and anions, gives rise to significant modulation of absorption and emission spectral properties. Using the absorption and emission spectral outputs toward specific anions and cations, we can demonstrate combinatorial Boolean logic functions of AND, OR, and NOT gates and the keypad lock. Additionally, fuzzy logic is employed to fabricate an infinite-valued setup to identify the indefinite values in between true (1) and false (0) states. ANN- and ANFIS-based modeling approaches were also employed using different combinations of inputs and output data. The results show that fuzzy, ANN, and ANFIS can quite accurately predict the experimental data. The statistical performance indicators (such as MSE and RMSE) indicate that the predicted values of the sensing data (absorption and emission spectral outputs) by ANFIS models are comparable to the experimental data. Therefore, the adopted computational intelligence-based approach can be considered a potential ion sensing data model for tpy-HImzPh_3_.

## Data Availability

The original contributions presented in the study are included in the article/Supplementary Material, further inquiries can be directed to the corresponding author.
