# Supplementary material for: Fuzzy Logic, Artificial Neural Network, and Adaptive Neuro-Fuzzy Inference Methodology for Soft Computation and Modeling of Ion Sensing Data of a Terpyridyl-Imidazole Based Bifunctional Receptor
Source: Front Chem. 2022 Mar 23;10:864363. doi: 10.3389/fchem.2022.864363 (PMC8984201; doi:10.3389/fchem.2022.864363)
Supplement: Supplementary file 1 [file DataSheet1.PDF]

Supplementary Material

Fuzzy Logic, Artificial Neural Network and Adaptive Neuro-Fuzzy Inference Methodology for Soft Computation and Modeling of Ion Sensing Data of a Terpyridyl-Imidazole Based Bifunctional Receptor

Anik Sahoo and Sujoy Baitalik*

Inorganic Chemistry Section, Department of Chemistry, Jadavpur University

Kolkata 700 032, India

Table S1. Rules for the Fuzzy Logic System by taking input 1 (F^-) and input 2 (H^+) as the Inputs and emission intensity as the Output. The Rules Consist of the Following Statements.

1. If (input1 is vl) and (input2 is vl) then (output1 is vh) (1)
2. If (input1 is vl) and (input2 is l) then (output1 is vh) (1)
3. If (input1 is vl) and (input2 is l) then (output1 is vh) (1)
4. If (input1 is vl) and (input2 is m) then (output1 is vh) (1)
5. If (input1 is vl) and (input2 is h) then (output1 is vh) (1)
6. If (input1 is vl) and (input2 is vh) then (output1 is vh) (1)
7. If (input1 is l) and (input2 is vl) then (output1 is h) (1)
8. If (input1 is l) and (input2 is l) then (output1 is h) (1)
9. If (input1 is l) and (input2 is m) then (output1 is vh) (1)
10. If (input1 is l) and (input2 is h) then (output1 is vh) (1)
11. If (input1 is l) and (input2 is vh) then (output1 is vh) (1)
12. If (input1 is m) and (input2 is vl) then (output1 is m) (1)
13. If (input1 is m) and (input2 is l) then (output1 is m) (1)
14. If (input1 is m) and (input2 is m) then (output1 is m) (1)
15. If (input1 is m) and (input2 is h) then (output1 is h) (1)
16. If (input1 is m) and (input2 is h) then (output1 is h) (1)
17. If (input1 is m) and (input2 is vh) then (output1 is h) (1)
18. If (input1 is h) and (input2 is vl) then (output1 is vl) (1)
19. If (input1 is h) and (input2 is l) then (output1 is vl) (1)
20. If (input1 is h) and (input2 is m) then (output1 is m) (1)
21. If (input1 is h) and (input2 is h) then (output1 is m) (1)
22. If (input1 is h) and (input2 is vh) then (output1 is h) (1)
23. If (input1 is vh) and (input2 is vl) then (output1 is vl) (1)
24. If (input1 is vh) and (input2 is l) then (output1 is vl) (1)
25. If (input1 is vh) and (input2 is l) then (output1 is vl) (1)
26. If (input1 is vh) and (input2 is m) then (output1 is l) (1)
27. If (input1 is vh) and (input2 is h) then (output1 is m) (1)
28. If (input1 is vh) and (input2 is vh) then (output1 is m) (1)
29. If (input1 is vl) then (output1 is vh) (1)
30. If (input1 is l) then (output1 is h) (1)
31. If (input1 is m) then (output1 is m) (1)
32. If (input1 is h) then (output1 is l) (1)
33. If (input1 is vh) then (output1 is vl) (1)
34. If (input2 is vl) then (output1 is vh) (1)
35. If (input2 is l) then (output1 is vh) (1)
36. If (input2 is m) then (output1 is vh) (1)
37. If (input2 is h) then (output1 is vh) (1)
38. If (input2 is vh) then (output1 is vh) (1)

Table S2. Values of emission intensity as a function of $n_{F^-}/n_{tpy\text{-}HImzPh3}$ and $n_{H^+}/n_{tpy\text{-}HImzPh3}$.

Input 1 (F^-)	Input 2 (H^+)	Emission Intensity at 485nm
0	0	712
0	1	714
1	0	534
2	0	100
3	0	70
4	0	6
0	2	715
0	3	716
0	4	716
1	1	620
2	2	235
3	3	310
4	4	212
1	2	510
1	3	700
1	4	710
2.5	2	210
1	3.5	705
3.5	1	90
1.5	1.5	605
3	1.5	498
1	1.5	570
2	1	276
3	4	110
0.5	4	706

Table S3. Rules for the Sugeno method by taking input 1 (F^-) and input 2 (H^+) as the Inputs and emission intensity as the Output. The Rules Consist of the Following Statements.

1. If (input1 is in1mf1) and (input2 is in2mf1) then (output is out1mf1) (1)
2. If (input1 is in1mf1) and (input2 is in2mf2) then (output is out1mf2) (1)
3. If (input1 is in1mf1) and (input2 is in2mf3) then (output is out1mf3) (1)
4. If (input1 is in1mf1) and (input2 is in2mf4) then (output is out1mf4) (1)
5. If (input1 is in1mf1) and (input2 is in2mf5) then (output is out1mf5) (1)
6. If (input1 is in1mf2) and (input2 is in2mf1) then (output is out1mf6) (1)
7. If (input1 is in1mf2) and (input2 is in2mf2) then (output is out1mf7) (1)
8. If (input1 is in1mf2) and (input2 is in2mf3) then (output is out1mf8) (1)
9. If (input1 is in1mf2) and (input2 is in2mf4) then (output is out1mf9) (1)
10. If (input1 is in1mf2) and (input2 is in2mf5) then (output is out1mf10) (1)
11. If (input1 is in1mf3) and (input2 is in2mf1) then (output is out1mf11) (1)
12. If (input1 is in1mf3) and (input2 is in2mf2) then (output is out1mf12) (1)
13. If (input1 is in1mf3) and (input2 is in2mf3) then (output is out1mf13) (1)
14. If (input1 is in1mf3) and (input2 is in2mf4) then (output is out1mf14) (1)
15. If (input1 is in1mf3) and (input2 is in2mf5) then (output is out1mf15) (1)
16. If (input1 is in1mf4) and (input2 is in2mf1) then (output is out1mf16) (1)
17. If (input1 is in1mf4) and (input2 is in2mf2) then (output is out1mf17) (1)
18. If (input1 is in1mf4) and (input2 is in2mf3) then (output is out1mf18) (1)
19. If (input1 is in1mf4) and (input2 is in2mf4) then (output is out1mf19) (1)
20. If (input1 is in1mf4) and (input2 is in2mf5) then (output is out1mf20) (1)
21. If (input1 is in1mf5) and (input2 is in2mf1) then (output is out1mf21) (1)
22. If (input1 is in1mf5) and (input2 is in2mf2) then (output is out1mf22) (1)
23. If (input1 is in1mf5) and (input2 is in2mf3) then (output is out1mf23) (1)
24. If (input1 is in1mf5) and (input2 is in2mf4) then (output is out1mf24) (1)
25. If (input1 is in1mf5) and (input2 is in2mf5) then (output is out1mf25) (1)

Table S4. Values of absorption intensity as a function of $n_{Fe2+}/n_{tpy\text{-}HImzPh3}$ and $n_{F-}/n_{tpy\text{-}HImzPh3}$.

Input 1 (Fe^{2+})	Input 2 (F^-)	Experimental output Data
0	0	0
5	0	0.53
0	13	0.01
1	5	0.24
2	6	0.21
3	7	0.19
4	8	0.18
5	9	0.15
1	10	0.011
2	11	0.005
3	12	0.001
5	13	0.0001
5	5	0.38
2.5	8	0.15
1.5	8	0.09
3.5	10	0.07

Table S5. Rules for the Fuzzy Logic System by taking input 1 (Fe^{2+}) and input 2 (F^-) as the Inputs and absorption intensity as the Output. The Rules Consist of the Following Statements.

1. If (input1 is L) then (output1 is L) (1)
2. If (input1 is M) then (output1 is M) (1)
3. If (input2 is L) then (output1 is L) (1)
4. If (input2 is M) then (output1 is L) (1)
5. If (input2 is H) then (output1 is L) (1)
6. If (input1 is H) then (output1 is H) (1)
7. If (input1 is L) and (input2 is L) then (output1 is L) (1)
8. If (input1 is L) and (input2 is M) then (output1 is L) (1)
9. If (input1 is L) and (input2 is H) then (output1 is L) (1)
10. If (input1 is M) and (input2 is L) then (output1 is L) (1)
11. If (input1 is M) and (input2 is M) then (output1 is L) (1)
12. If (input1 is M) and (input2 is H) then (output1 is L) (1)
13. If (input1 is H) and (input2 is L) then (output1 is H) (1)
14. If (input1 is H) and (input2 is M) then (output1 is M) (1)
15. If (input1 is H) and (input2 is H) then (output1 is L) (1)

Table S6. Rules for the Sugeno method by taking input 1 (Fe^{2+}) and input 2 (F^-) as the Inputs and absorption intensity as the Output. The Rules Consist of the Following Statements.

1. If (input1 is in1mf1) and (input2 is in2mf1) then (output is out1mf1) (1)
2. If (input1 is in1mf1) and (input2 is in2mf2) then (output is out1mf2) (1)
3. If (input1 is in1mf1) and (input2 is in2mf3) then (output is out1mf3) (1)
4. If (input1 is in1mf2) and (input2 is in2mf1) then (output is out1mf4) (1)
5. If (input1 is in1mf2) and (input2 is in2mf2) then (output is out1mf5) (1)
6. If (input1 is in1mf2) and (input2 is in2mf3) then (output is out1mf6) (1)
7. If (input1 is in1mf3) and (input2 is in2mf1) then (output is out1mf7) (1)
8. If (input1 is in1mf3) and (input2 is in2mf2) then (output is out1mf8) (1)
9. If (input1 is in1mf3) and (input2 is in2mf3) then (output is out1mf9) (1)

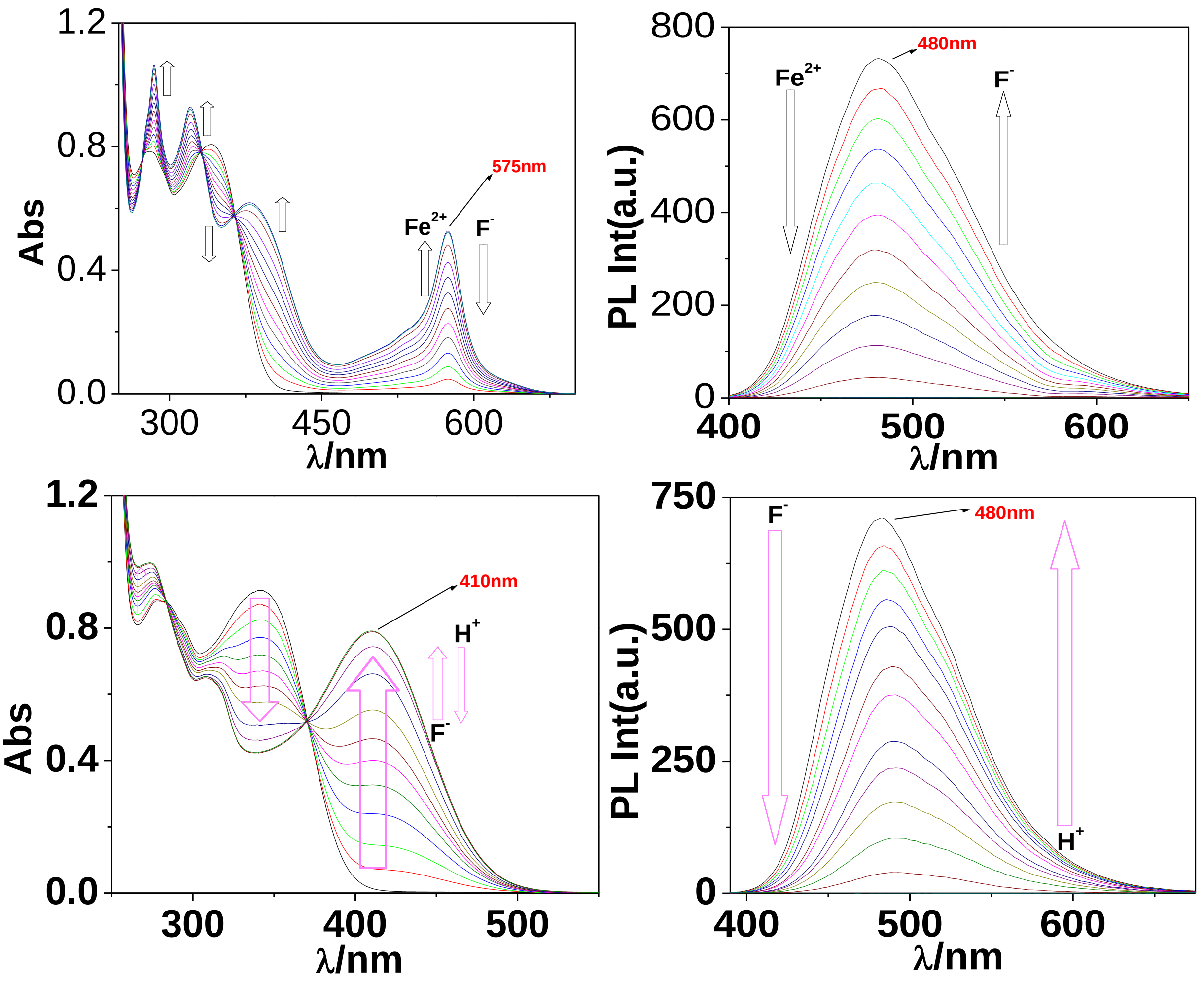

Figure S1. UV-vis absorption and emission spectrum of tpy-HImzPh$_3$ upon incremental addition of Fe^{2+} followed by F^- (a and b, respectively), while figure c and d represent the absorption and emission of tpy-HImzPh$_3$ upon incremental addition of F^- followed by H^+ in DMF-MeCN, 1:9, v/v).

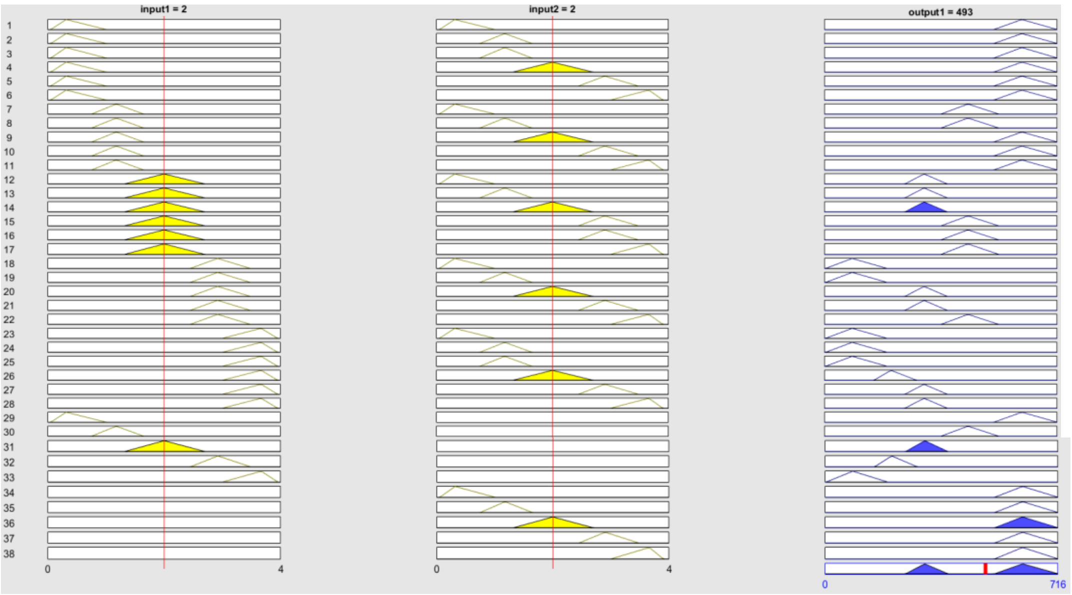

Figure S2. Mamdani rule view for tpy-HImzPh$_3$.

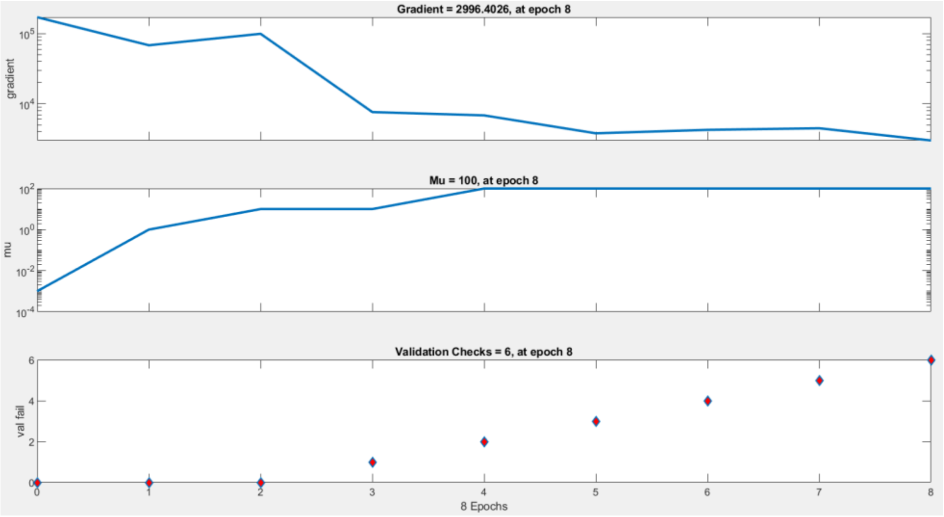

Figure S3. Training state of the ANN model up to epoch 8.

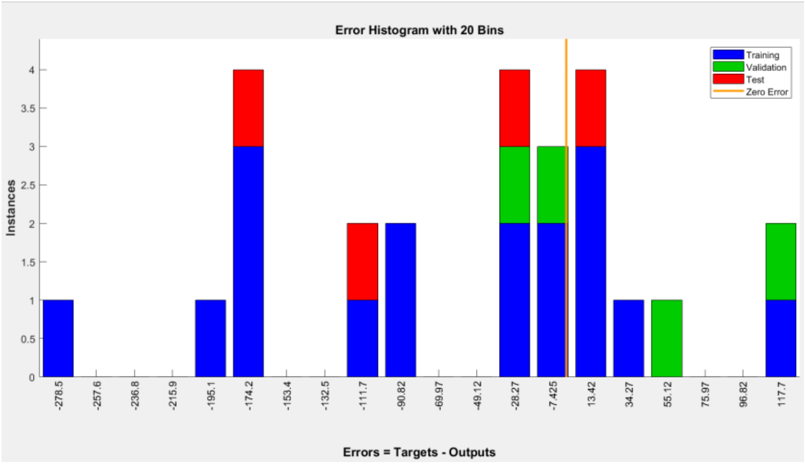

Figure S4. Error histogram in ANN model training process.

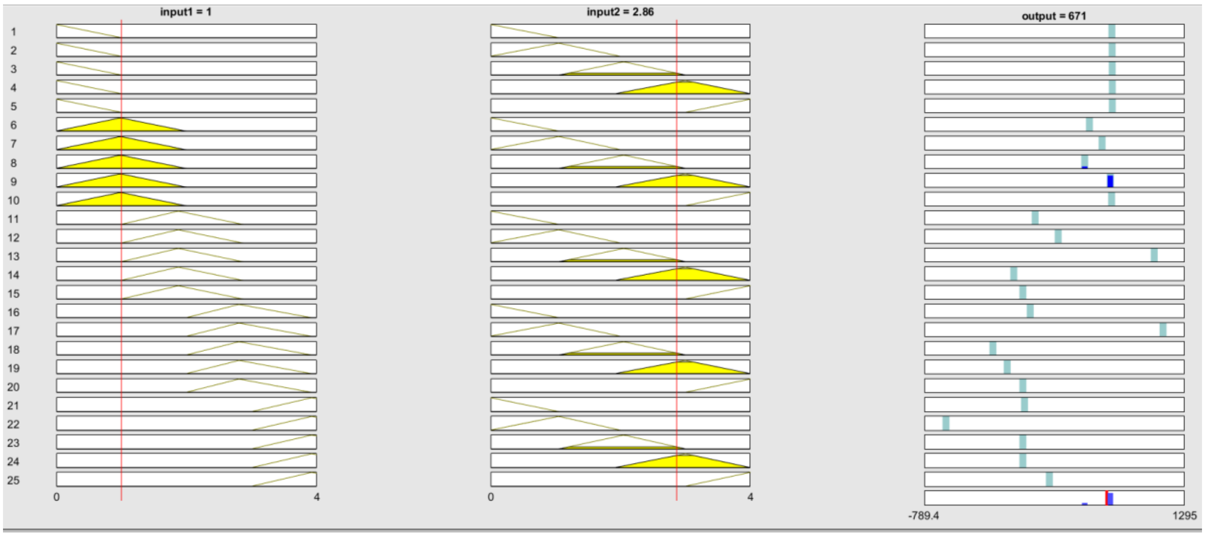

Figure S5. Sugeno rule view for tpy-HImzPh$_3$**.**

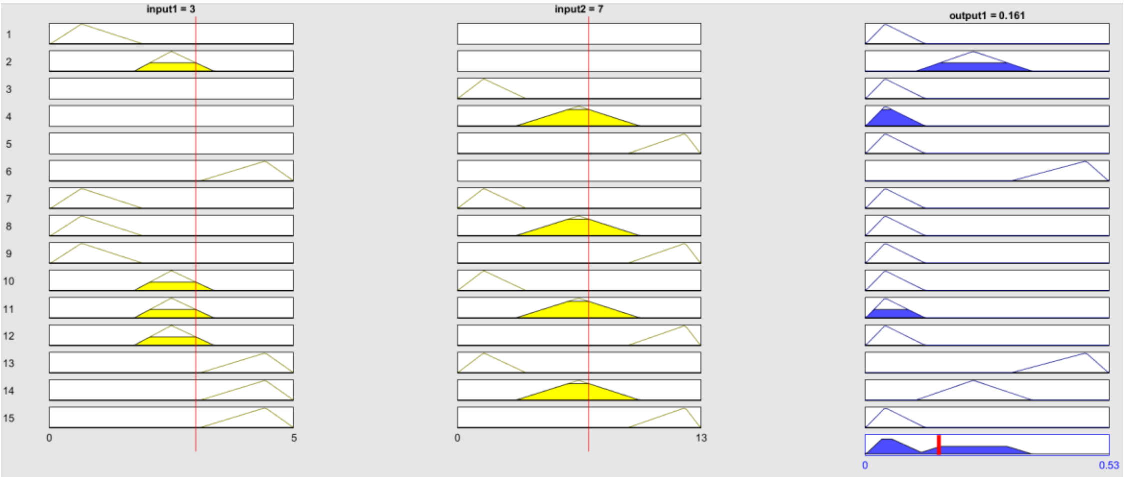

Figure S6. Mamdani rule view for tpy-HIm

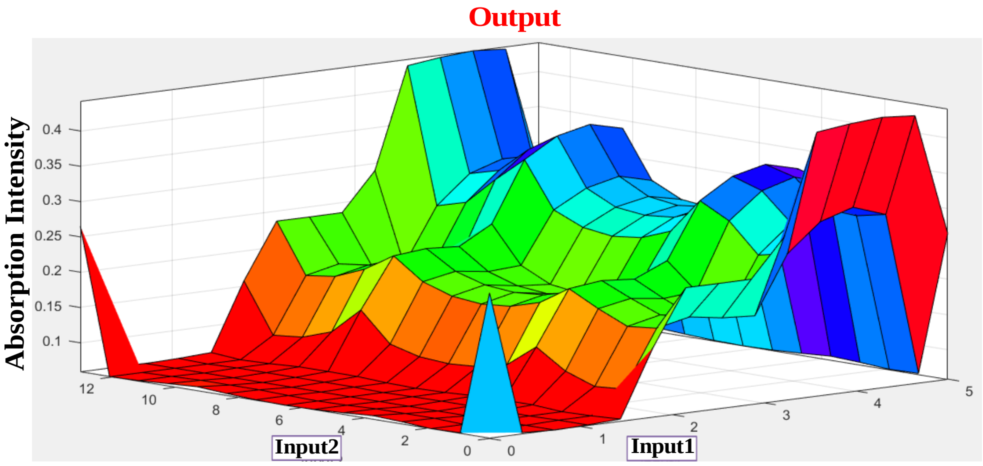

Figure S7. Three-dimensional representation of the dependence of absorption intensity of tpy-HImzPh$_3$ at 575nm as a function of simultaneous injection of two chemical inputs (Fe^{2+} and F^-).

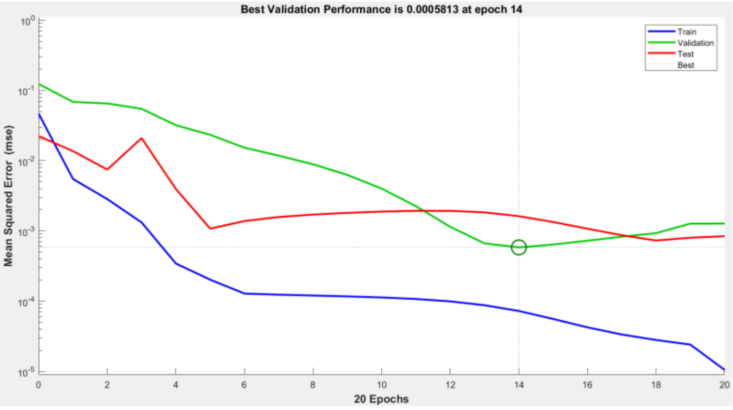

Figure S8. Performance of the designed ANN model.

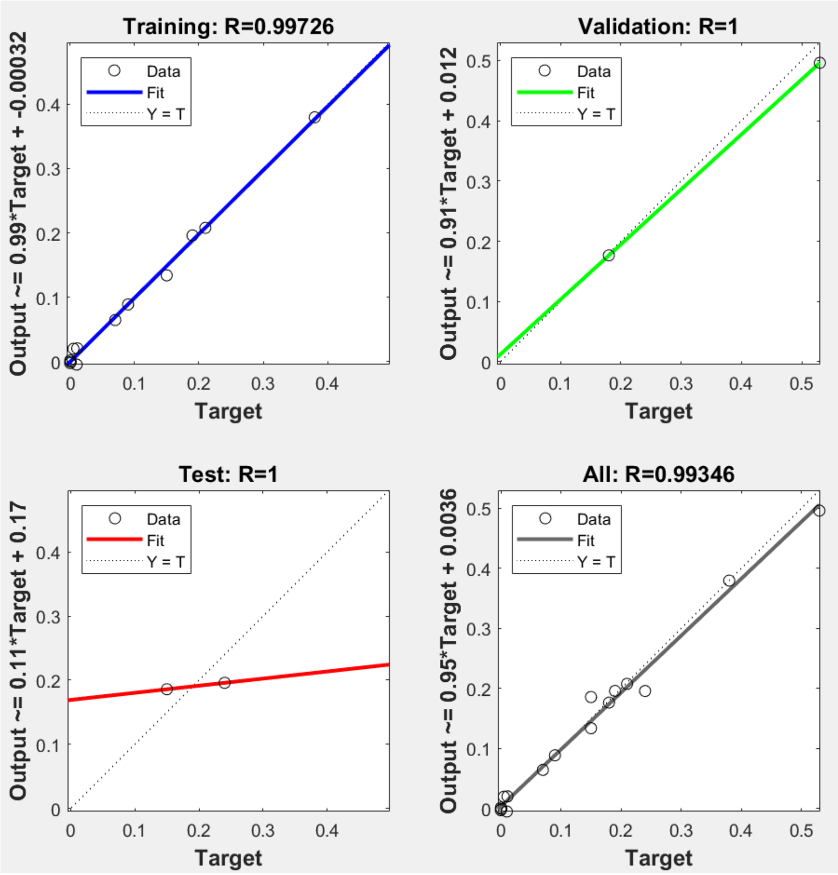

Figure S9. Regression data of the ANN model.

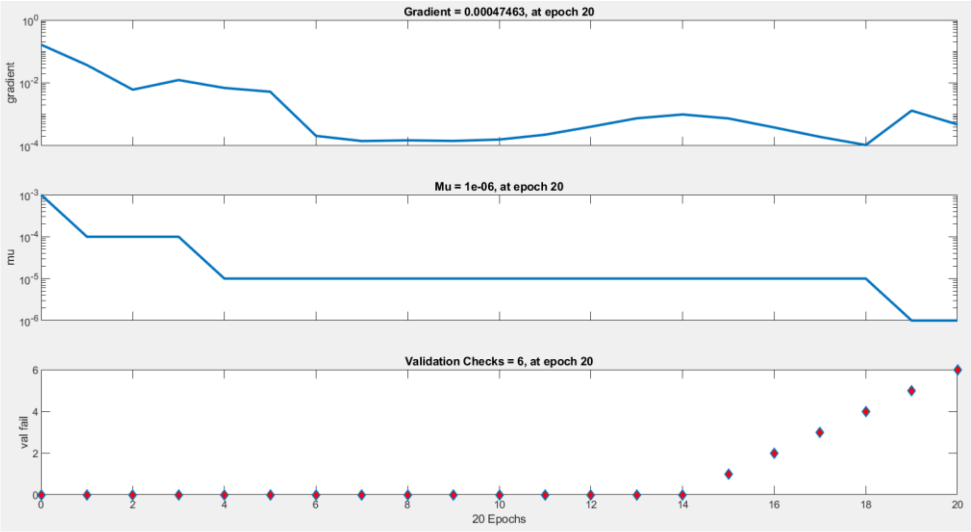

Figure S10. Training state of the ANN model up to epoch 20.

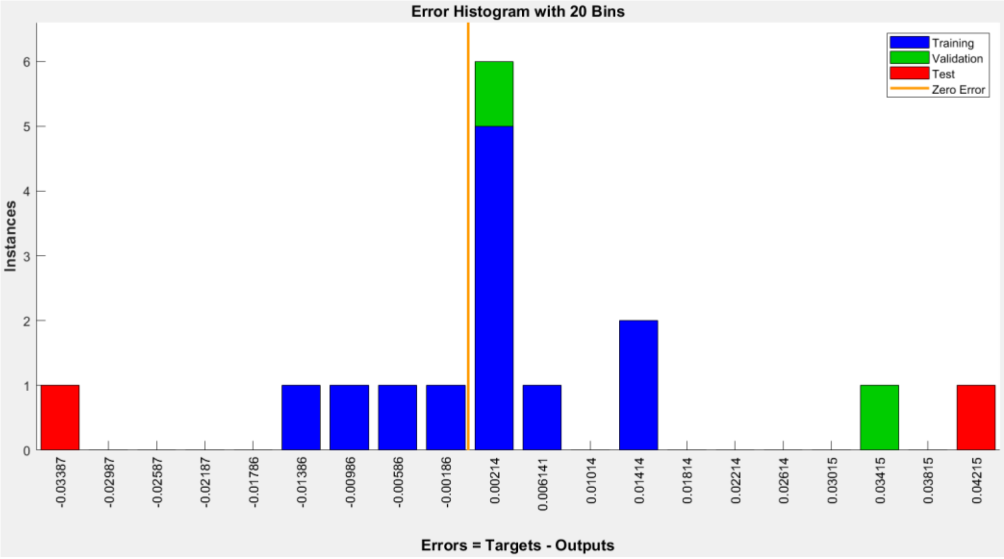

Figure S11. Error histogram of the ANN model.

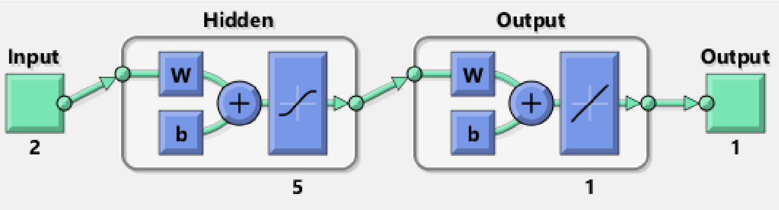

Figure S12. Artificial neural network model consisting of 5 hidden layer.

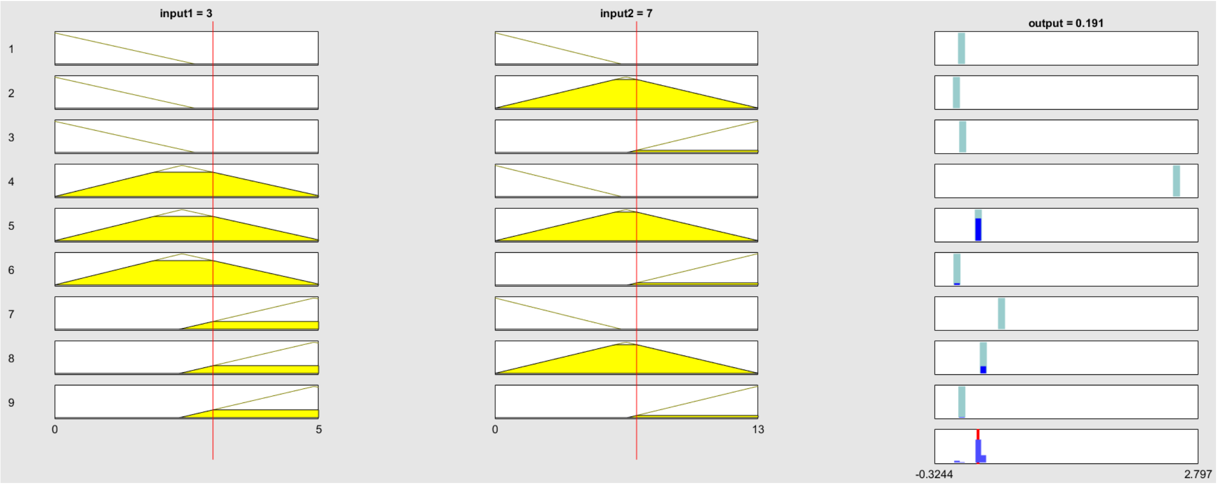

Figure S13. Sugeno rule view for tpy-HImzPh$_3$.

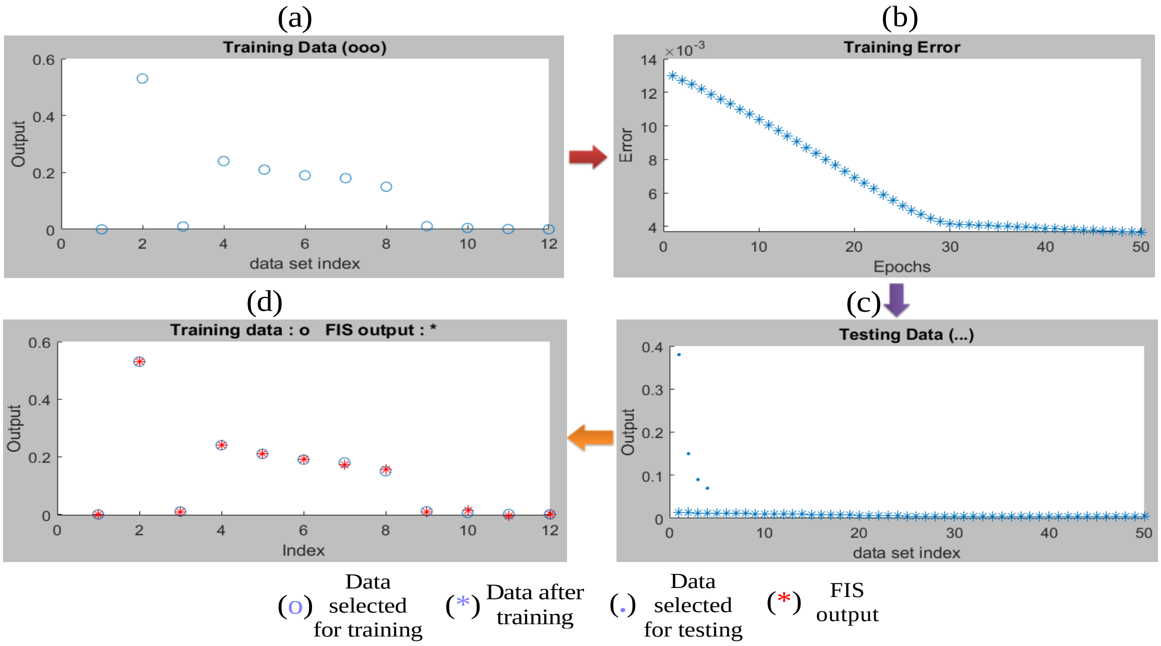

Figure S14. (a) Selected training data to design anfis model. (b) Training error minimization up to 50 epochs. (c) Combination of training and testing data. (d) Compilation of testing data and FIS output.

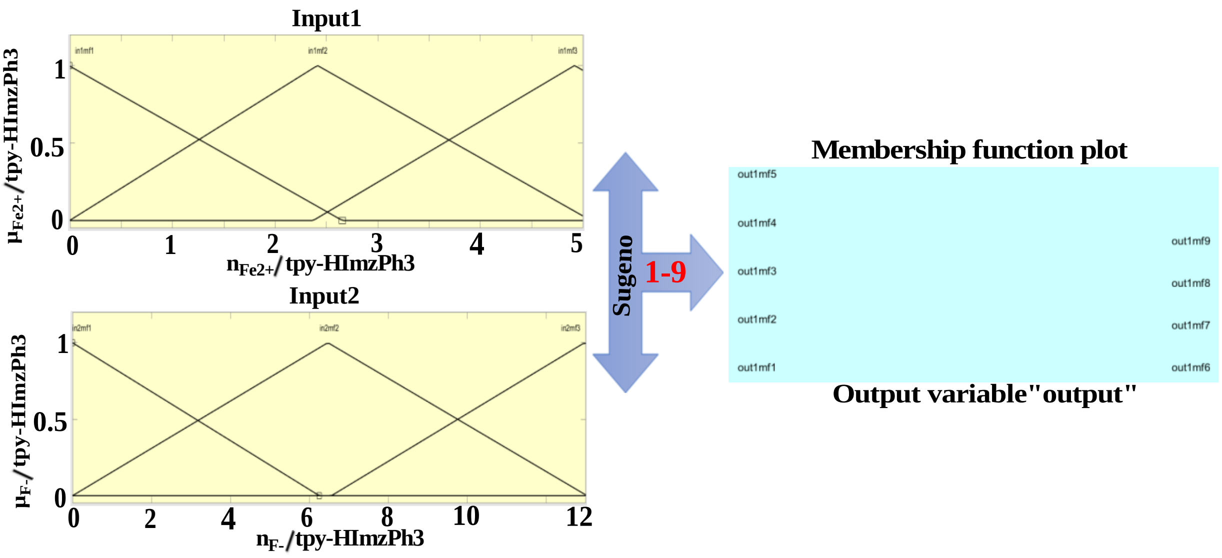

Figure S15. Schematic presentation of ANFIS based on sugeno method maintaining 9 rules.

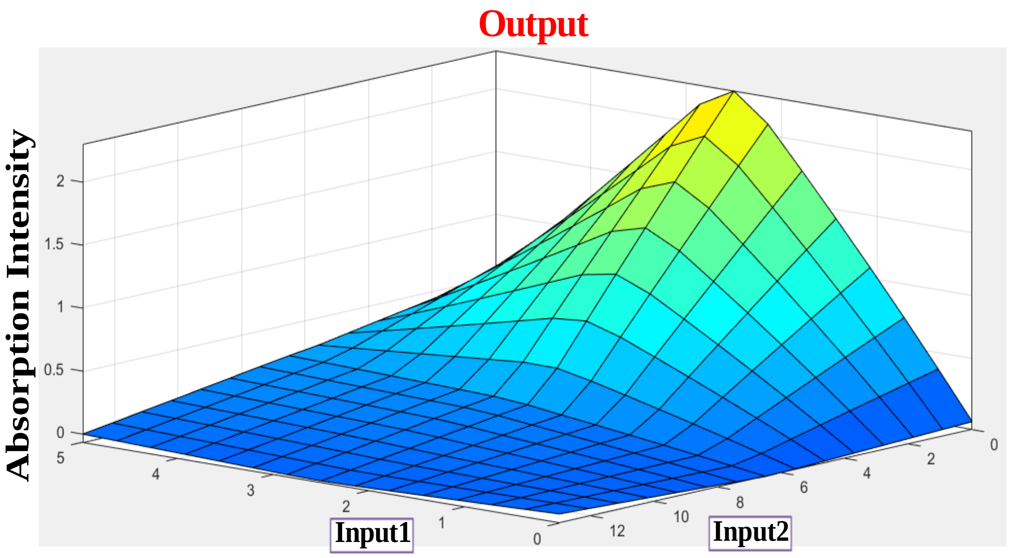

Figure S16. Three-dimensional representation (based on Sugeno method) of the dependence of absorption intensity of tpy-HImzPh$_3$ at 575nm as a function of simultaneous injection of two chemical inputs (Fe^{2+} and F^-).

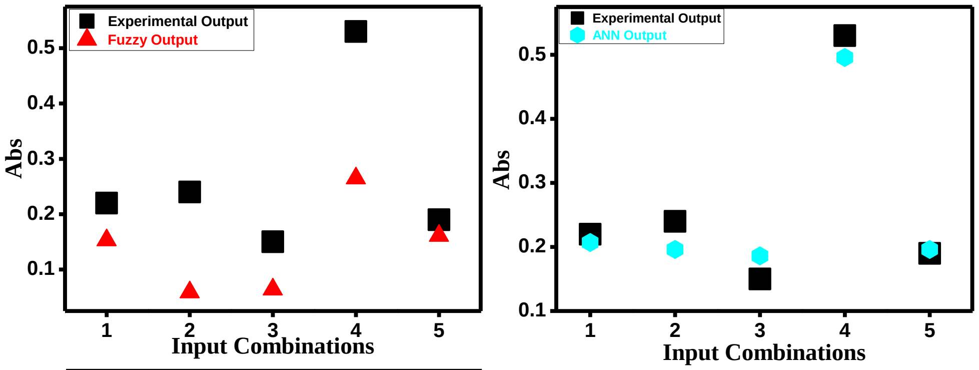

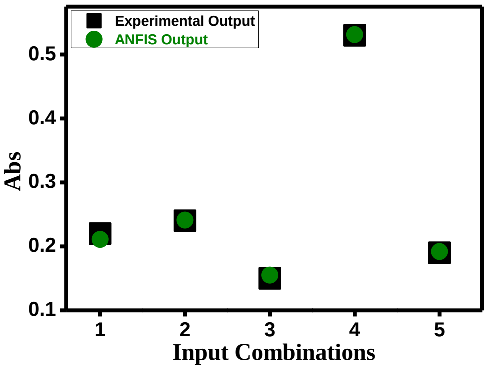

Input Combinations	Fe^{2+}	F^{-}
1	1	1
2	4	4
3	4	0
4	1	3
5	1	2

Figure S17. Comparison between experimental absorption output data and Fuzzy, ANN and ANFIS output data.
